# Connecting the dots: Managing species interaction networks to mitigate the impacts of global change

**DOI:** 10.7554/eLife.98899

**Published:** 2025-04-08

**Authors:** Luis Abdala-Roberts, Adriana Puentes, Deborah L Finke, Robert J Marquis, Marta Montserrat, Erik H Poelman, Sergio Rasmann, Arnaud Sentis, Celia C Symons, Nicole M van Dam, Gina Wimp, Christer Björkman, Kailen A Mooney

**Affiliations:** 1 Departamento de Ecología Tropical, Campus de Ciencias Biologicas y Agropecuarias, Universidad Autonoma de Yucatán Yucatan Mexico; 2 Department of Ecology, Swedish University of Agricultural Sciences Uppsala Sweden; 3 Division of Plant Sciences, University of Missouri Columbia United States; 4 Department of Biology and the Whitney R. Harris World Ecology Center, University of Missouri–St. Louis St. Louis United States; 5 Instituto de Hortofruticultura Subtropical y Mediterránea “La Mayora” (IHSM-UMA-CSIC), Consejo Superior de Investigaciones Cientıficas Málaga Spain; 6 Laboratory of Entomology, Wageningen University Wageningen Netherlands; 7 Institute of Biology, University of Neuchȃtel Neuchâtel Switzerland; 8 UMR RECOVER, INRAE, Aix Marseille University Aix-en-Provence France; 9 Department of Ecology and Evolutionary Biology, University of California, Irvine Irvine United States; 10 Plant Biotic Interactions, Leibniz Institute for Vegetable and Ornamental Crops Grosbeeren Germany; 11 Department of Biology, Georgetown University Washington, DC United States; University of Zurich Switzerland; University of Zurich Switzerland

**Keywords:** global change, species interactions, conservation, biodiversity, ecosystem service, food webs

## Abstract

Global change is causing unprecedented degradation of the Earth’s biological systems and thus undermining human prosperity. Past practices have focused either on monitoring biodiversity decline or mitigating ecosystem services degradation. Missing, but critically needed, are management approaches that monitor and restore species interaction networks, thus bridging existing practices. Our overall aim here is to lay the foundations of a framework for developing network management, defined here as the study, monitoring, and management of species interaction networks. We review theory and empirical evidence demonstrating the importance of species interaction networks for the provisioning of ecosystem services, how human impacts on those networks lead to network rewiring that underlies ecosystem service degradation, and then turn to case studies showing how network management has effectively mitigated such effects or aided in network restoration. We also examine how emerging technologies for data acquisition and analysis are providing new opportunities for monitoring species interactions and discuss the opportunities and challenges of developing effective network management. In summary, we propose that network management provides key mechanistic knowledge on ecosystem degradation that links species- to ecosystem-level responses to global change, and that emerging technological tools offer the opportunity to accelerate its widespread adoption.

## Introduction

**Figure 1.**
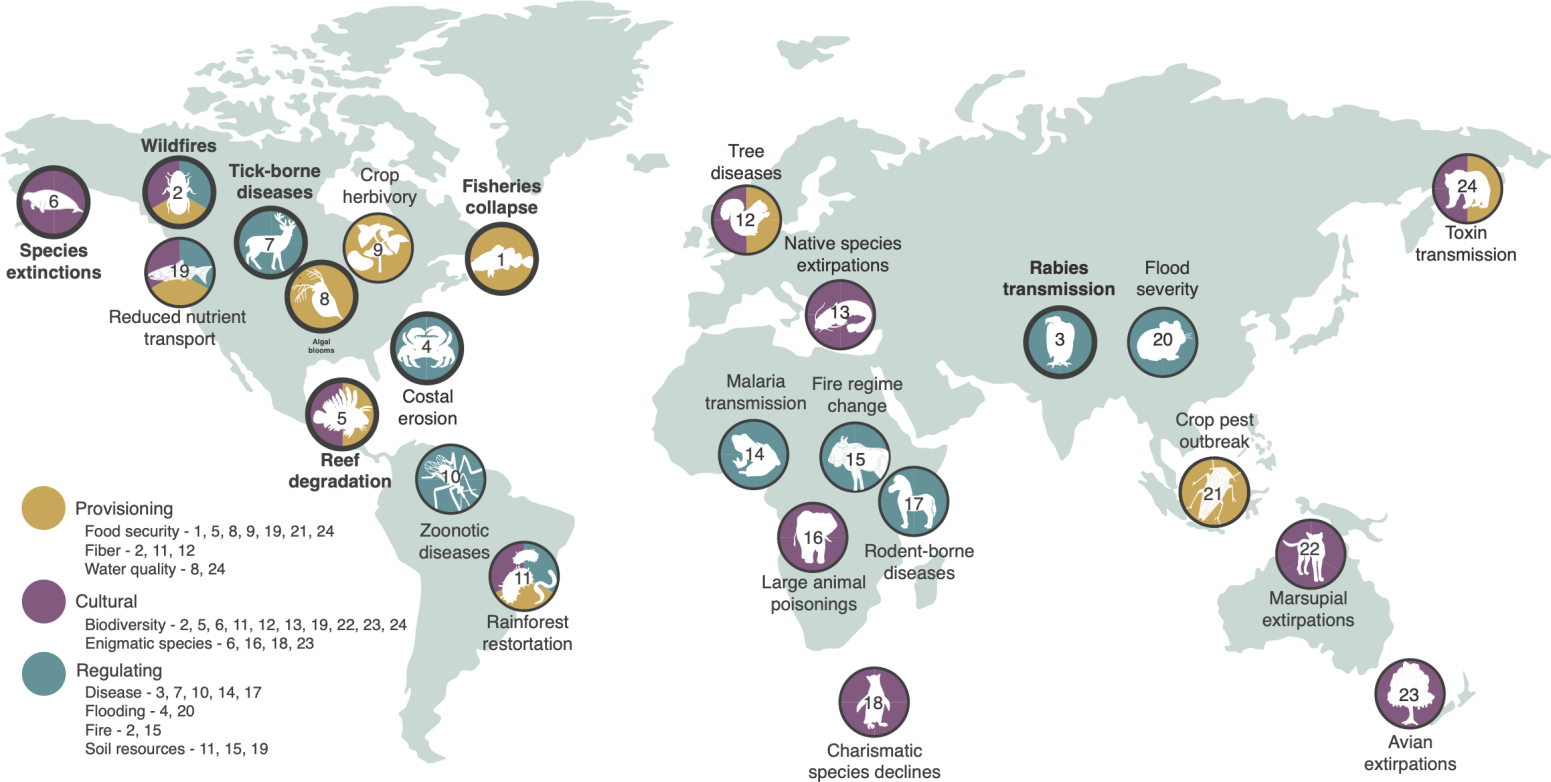
Examples of global change altering networks of species interactions, impacting key species or groups of species that provide ecosystem services. Four descriptors are provided for each case study: (1) the disrupted ecosystem service is listed (icon text); (2) colors depict the class or classes of ecosystem services (provisioning, cultural, or regulating); (3) an icon depicts the key species (or group of species) that is the ecosystem service (or dis-service) provider driving the effect; and (4) numbers refer to network diagrams showing how altered interaction networks resulted in the disruption of the ecosystem service. Highlighted case studies (bold text and circles) are described in text below with network diagrams provided in Figure 3, representing case studies that provide provisioning services (#1, #2), regulating services (#3, #4), and cultural services (#5, #6), as well as in Figure 4, representing case studies of successful network management (#7, #8). Explanations of other case studies (descriptions, network diagrams) are provided in Appendix 1. Where case studies address multiple classes of ecosystem service, subsequent figures (Figure 3 and Appendix 1) highlight a single class.

Humans are having unprecedented effects on the Earth’s biological systems (Figure 1). Climate change affects even the most remote and protected ecosystems, while natural habitats are destroyed and degraded through land-use practices, altered nutrient supply, toxic contamination, invasive species, noise and light pollution, fragmentation, and other pernicious threats (Folke et al., 2021). These global change drivers are jeopardizing the ecosystem services upon which human prosperity depends (Millennium Ecosystem Assessment, 2005) and impact biodiversity through both species’ extinctions (Johnson et al., 2017; Díaz et al., 2019) and the redistribution of species into novel biotic communities (Pejchar and Mooney, 2009; Pecl et al., 2017). Human health and wellbeing are at risk and inextricably dependent upon our ability to understand and mitigate global change impacts on biological systems (Intergovernmental Science-Policy Platform on Biodiversity and Ecosystem Services, 2024).

**Figure 2.**
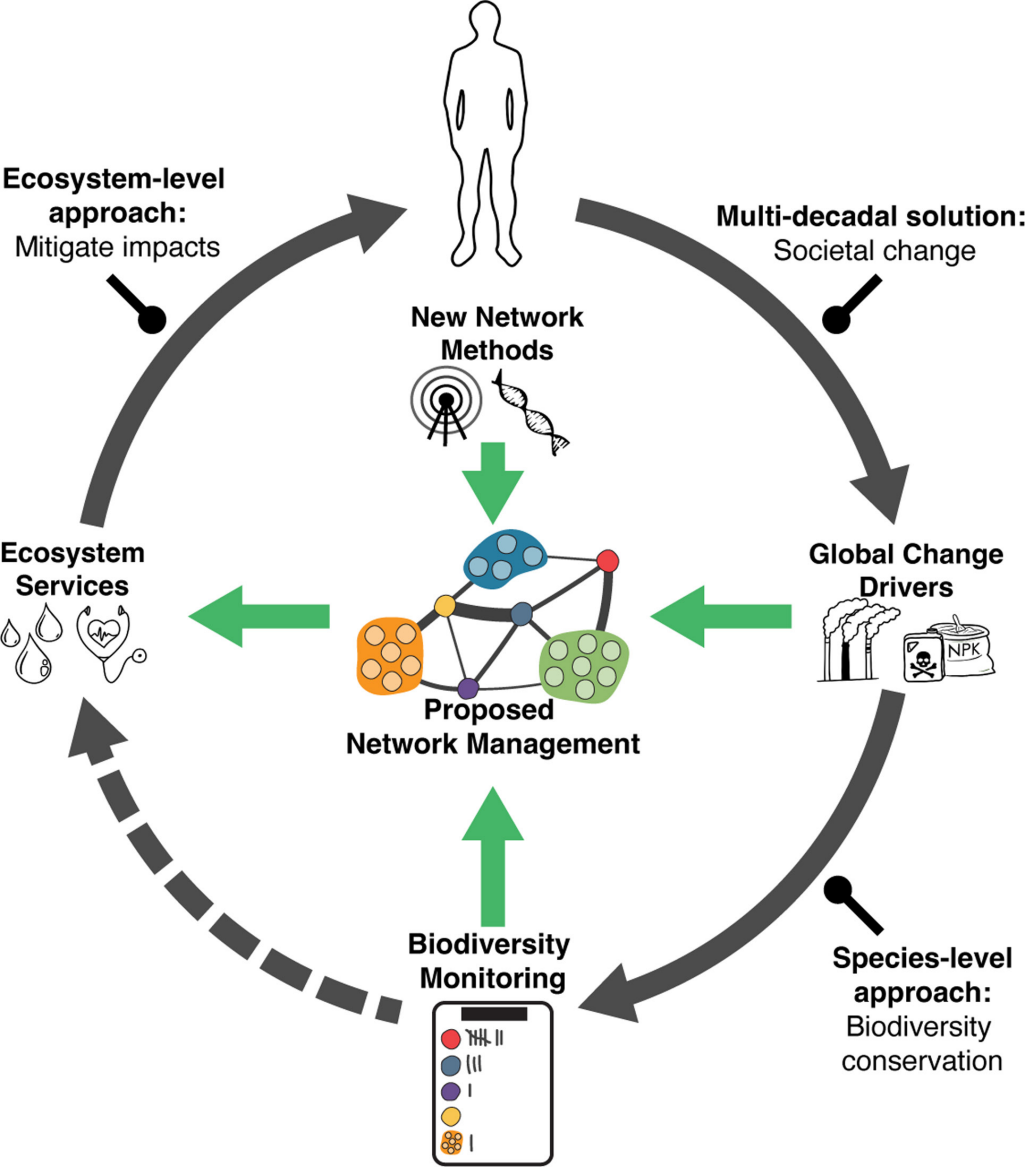
Linkages between global change drivers, societal responses to their impacts, ecosystem services, and human prosperity and wellbeing. Global change drivers (e.g., CO_2_, fertilizers, other pollutants) are recognized to affect both biodiversity (e.g., endangered species), and the ecosystem services (e.g., air and water quality) that underlie human wellbeing (solid/curved/gray arrows). Societal responses to these impacts include multi-decadal efforts to transform to sustainable socio-economic systems, biodiversity conservation, or restoration, and attempts to mitigate the impacts of degraded ecosystem services on humans (straight/rounded head/black arrows). While biodiversity monitoring has been an important component of conservation, there has been little connection between such efforts and the restoration of ecosystem services (dashed/curved/gray arrow). We propose the need to develop a third approach, termed network management (green arrows) using information on species interactions and the networks that emerge from them which drive the performance of key ecosystem providers. Such an approach can provide a mechanistic understanding of how global change impacts on species scale up to affect ecosystem services and in turn inform effective management solutions. **Figure center**: A schematic representation of an interaction network (aka *food web*). Individual circles represent species, lines represent interactions, and line thickness the strengths of interactions. Networks can be depicted and studied with links between individual species (single circles) or between groups of species (trophic levels or guilds; clusters of circles) that have a similar function within an ecosystem. All species within a network are ultimately linked by some combination of direct and indirect interactions.

Efforts to understand and mitigate human effects on biological systems have focused on two approaches (Figure 2). On the one hand, species-level approaches have been based on biological monitoring to document changes in species presence or abundances, as well as the aggregate effects on overall biodiversity locally or regionally. Such efforts are motivated by conservation laws and agreements (e.g., the Endangered Species Act (in the US) and the International Union for Conservation of Nature’s Red List) and the ethical imperative to avoid species extinctions, including bioindicator species that are monitored as surrogates for numerous unmonitored species (Lawton and Gaston, 2001). These efforts have been crucial in alerting us to the severity of the on-going biodiversity crisis of species decline and extinction in many regions (Lindenmayer and Likens, 2010), but often do not reveal the mechanistic basis of these changes.

On the other hand, ecosystem-level approaches (Figure 2) are based on legally mandated monitoring efforts focused on focal ecosystem services (Lovett et al., 2007) (e.g., the US Clean Air and Water Acts, EU Ambient Air and Water Framework Directives). Technologically sophisticated surveillance programs have been developed to predict such effects, including the detection and prediction of wildfires, coastal and inland flooding, human disease vectors, and agricultural diseases and pests. At this level, responses have focused on mitigating the human consequences of degraded services but do not address the root cause or ecological mechanisms by which global change impacts ecosystems. Furthermore, the impacts of global change on ecosystem services often operate through complex, indirect pathways resulting in so-called ‘ecological surprises’ (Paine et al., 1998; Filbee‐Dexter et al., 2017), the unexpected nature of which undermine effective planning and mitigation.

Missing, and critically needed, are practices that link these two approaches, documenting the mechanisms by which global change impacts on individual species or groups of species scale up to produce emergent impacts on ecosystem services. Acquiring this knowledge is urgent, as it is the basis for the development of nature-based solutions to protect and restore the Earth’s biological systems and protect human prosperity.

In this paper, we propose that ecological network management – defined here as the study, monitoring, and management of species interactions (Figure 2) – provides the mechanistic knowledge needed to bridge existing species- and ecosystem-level practices. We first review theory on species interactions, food web network structure, and ecosystem services predicting that disrupted species interactions should drive the degradation of natural systems to the detriment of human wellbeing. We then examine case studies from ecosystems across the planet showing how the disruption of species interaction networks underlies the degradation of ecosystem services. In turn, we present case studies demonstrating how the management (e.g., conservation or restoration) of networks has successfully mitigated such effects or restored degraded ecosystems. Finally, we go on to consider how novel and accessible technologies for data acquisition and analysis provide unprecedented opportunities for ecological network management, but also the logistical and regulatory challenges to implementing such programs. Our overarching goal is to lay out a general framework for developing network management.

Several important messages arise from this review: the drivers of global change act through complex mechanisms. Nevertheless, novel approaches to identify and monitor key interactions can reduce the likelihood of unwanted ecological surprises, efficiently guide management practices and, in so doing, provide nature-based solutions to protect and restore biodiversity and human prosperity.

### The importance of species interactions and networks

#### Interaction networks and indirect effects

A central goal in ecological science has been to understand how species interact, and the impacts these interactions have on ecosystems (Hairston et al., 1960; Paine, 1980). Sets of interactions can be depicted as networks (aka food webs), and can be described as occurring among individual species, or groups of species (Figure 1, center). These groups – commonly referred to as guilds – are based on the species trophic level (i.e., plants, herbivores, predators, decomposers) and functional roles within those trophic levels (e.g., fruit-eating vs. insect-eating birds; deciduous vs. perennial plants). Interaction strengths can vary, such that the change in abundance in one species can have a large (strong interaction) or small (weak interaction) effect on the abundance of another species. Most networks make use of data on the relative frequency of interactions (collected by observation) though there are examples with data on interaction strength (usually obtained via experimental approaches, e.g., species exclusions). In addition, multiple networks among subsets of species within communities can themselves be linked, forming networks of networks (Pocock et al., 2012; Timóteo et al., 2018).

Within networks, all species (and guilds) are ultimately connected not only by direct interactions, but also indirect interactions where species affect each other through their effects on one or more intermediate (transmitting) species. The loss, addition, or change in abundance (or elimination) of one species can thus affect an entire ecosystem due to a rippling cascade of indirect effects transmitted through altered interactions. The importance of indirect interactions is often unrecognized because they are difficult to observe under unperturbed conditions (Estes et al., 2011). However, ecological experiments and naturally occurring species invasions and extinctions have clearly demonstrated the ubiquity and large effects of indirect interactions on the abundance of individual species, as well as emergent ecosystem processes such as decomposition and cycles of water and nutrients (Wootton and Emmerson, 2005; Schmitz, 2008). Indeed, indirect effects can be stronger than direct effects, as the number of indirect links between species is far greater and because multiple indirect pathways may act synergistically (Estes et al., 2013).

#### Ecosystem services and key ecosystem service providers

Ecosystem services – the direct and indirect benefits that humans receive from natural systems (Millennium Ecosystem Assessment, 2005; Intergovernmental Science-Policy Platform on Biodiversity and Ecosystem Services, 2024) – can be placed within different categories. Provisioning services are resources humans draw directly from nature (e.g., food, wood and fiber, hydropower, biomass fuels, medicinal resources); cultural services are non-material benefits such as recreation, mental wellbeing, and spiritual sustenance; regulating services include the maintenance of ecosystem functions such as decomposition and carbon fixation as well as processes means by which nature delivers provisioning services (e.g., regulation of pollinator populations for food security). Regulating services also include the means by which ecosystem disservices are minimized or held in check (von Döhren and Haase, 2015), for example, mitigation of direct negative impacts on humans (e.g., animal attacks and disease) or the disruption of provisioning services (e.g., the negative impact of crop pests on food security). Finally, some classifications also include supporting services, that is, those that make it possible for ecosystems to provide the three primary services described above (e.g., primary production, soil formation, and habitat and biodiversity provision) (Millennium Ecosystem Assessment, 2005; Intergovernmental Science-Policy Platform on Biodiversity and Ecosystem Services, 2024). Although economic valuations of ecosystem services and disservices are difficult and often incomplete (Boerema et al., 2017), available estimates place their value globally in dozens of trillions of USD annually (Costanza et al., 2014).

#### The study of networks and linkages to ecosystem services

The study of interaction networks has followed three interrelated approaches. First, networks have been studied to understand the relative strength of different types of direct interactions, such as predator–prey or plant–herbivore interactions, or the relative importance of direct vs. indirect effects (Wootton and Emmerson, 2005; Estes et al., 2011). This body of work reveals that not all species (and guilds) are equivalent in terms of their effects on other species and ecosystem services (Bianco et al., 2024). Keystone species are relatively rare species that can have outsized effects on ecological communities, either due to their strong interactions with other species, or due to their roles as ecosystem engineers by altering the physical environment inhabited by other species (Paine, 1969; Jones et al., 1997). In addition, ecosystems are typically dominated by one or several foundation species that have large effects due to their high abundance (Ellison, 2019), including, for example, the relatively few coniferous tree species that dominate boreal and montane ecosystems in the northern hemisphere (Angelini et al., 2011).

Second, studies have also investigated the effects of biodiversity on species interactions and ecosystem function. Such work investigates how diversity within a guild of species (e.g., plants, pollinators, or predators) affects the guild’s interactions with other community members. There is strong evidence that diversity can determine both interaction strength and stability, defined as recovery to baseline conditions following disturbance (Tilman et al., 2014; Barnes et al., 2020; Buzhdygan et al., 2020). In some cases, the diversity within a guild can be more important than the abundance of the guild (number of individuals) in determining its effects on other components of an ecosystem (Nell et al., 2018).

Finally, network topology has been studied in depth, documenting all of the pairwise connections between two guilds of interacting species (e.g., plants and pollinators). The topologies are then assessed using network theory to understand the commonalities in properties and predict resilience to species loss (Tylianakis et al., 2008). Such work demonstrates that species vary dramatically in the number of interactions they engage with; typically, only a few species in a network are highly interactive, whereas most species engage in few interactions (Bascompte and Stouffer, 2009). The loss of these highly interactive species has the strongest effects on other community members and on the network as a whole (Memmott et al., 2004).

In summary, theory and empirical work on species interactions has demonstrated how keystone, foundation, and highly interactive species drive ecosystem functions and, unsurprisingly, make them key providers of ecosystem services (Kremen, 2005; Gray et al., 2014), and that the diversity within species guilds can be important both for interaction strength and stability.

### Global change acts in unexpected ways via altered species interactions

We next review case studies demonstrating how global change impacts on a relatively few key ecosystem providers can disrupt interaction networks and, in so doing, transmit and amplify effects to degrade ecosystem services (Tylianakis et al., 2008; Keyes et al., 2021; Bartomeus et al., 2021; Figure 1). These outcomes not only undermine human prosperity but are often unanticipated – so-called ecological surprises – because the underlying networks are typically unstudied.

#### Provisioning services

##### Food security – fisheries

**Figure 3.**
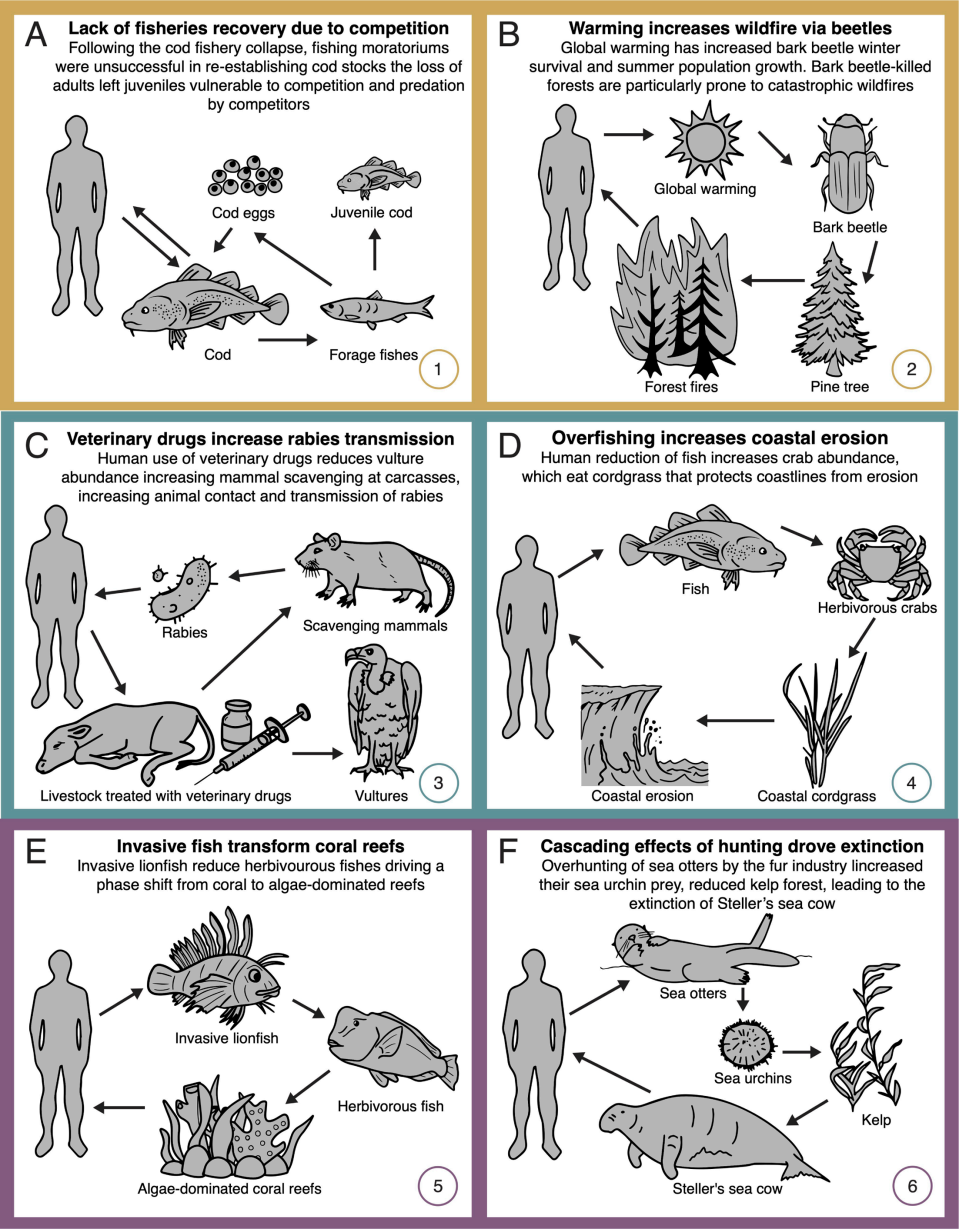
Network diagrams of global change impacts on (**A, B**) provisioning, (**C, D**) regulating, and (**E, F**) cultural ecosystem services through altered species interactions. See text for detailed explanations of each case study. Numbers (bottom right corners) reference the depiction of these case studies in Figure 1. Additional examples of degraded provisioning ecosystem services are provided in Appendix 1. Some case studies address multiple classes of ecosystem service (as shown in Figure 1), but individual classes are highlighted here and in Appendix 1.

Ocean fisheries represent an important source of food globally and provide huge economic benefits through employment both off and on shore. Like many other fisheries, Atlantic cod (*Gadus morhua*) populations collapsed in the early 1990s, devastating 39,000 fishers and reducing annual revenue by US $200 million (Myers et al., 1997). Surprisingly, cod populations have failed to recover despite extensive fishing moratoriums (Sguotti et al., 2019). We now know that this is because adult cod promote the survival of their own eggs and juveniles by keeping competitors and predators in check, thus maintaining adult stocks in a positive feedback loop. This dynamic was set in reverse when overharvesting of these protective adults indirectly increased the abundance of smaller fish species and invertebrates upon which adult cod had previously preyed (Frank et al., 2005). With the reduction in adult cod, predation on eggs and competition with juvenile cod increased (Frank et al., 2011; Figure 3A). As a result, adult cod stocks are now not only smaller, but also fluctuate dramatically, increasing economic uncertainty. As of yet, fisheries managers have been unable to restore historic interaction regimes in this system.

##### Forest resources

Forests provide timber, sequester carbon, and support clean water, biodiversity, and recreation services which collectively are estimated to be worth US $4.7 trillion annually (Costanza et al., 1997). Forest wildfires threaten these services. Paradoxically, the increase in catastrophic wildfires has been traced back to the indirect consequences of long-term fire suppression. By reducing tree mortality, decades of fire suppression have increased forest density and competition among trees, reducing tree vigor and thus vulnerability to attack from insects (Waring and Pitman, 1985; Figure 3B). Fire suppression also negatively affects predators that would otherwise suppress insect outbreaks (Hekkala et al., 2021). The indirect effects of fire suppression in turn increase tree mortality, leading to the accumulation of fuels for additional wildfires. At the same time, global warming has increased insect winter survival and accelerated summer population growth (Robbins et al., 2022). For example, bark beetles (Curculionidae) have affected 14 million hectares in western Canada (Safranyik et al., 2010), in some areas leading to the loss of >50% of the total merchantable pine (British Columbia Ministry of Forests, Lands and Natural Resource Operations, 2012). Because forests that are heavily infested by bark beetles are particularly prone to catastrophic wildfires (Lynch et al., 2006), wildfire frequencies are predicted to increase by 30–50% by 2100, resulting in a positive feedback loop in carbon production via reduced photosynthesis and increased carbon release associated with fires (United Nations Environment Programme, 2022).

### Regulating

#### Disease

Anthropogenic disturbances are altering disease dynamics worldwide, increasing transmission from animals to humans. Disease control strategies are frequently ineffective or even harmful due to a lack of understanding of ecological networks. For example, veterinary drugs for livestock unexpectedly increased human exposure to rabies. In Asia and Africa, the drug diclofenac unexpectedly rendered livestock corpses toxic to vultures (*Gyps* spp.), making those corpses accessible to scavenging mammals. In turn, mammal-to-mammal contact at corpses increased rabies transmission and ultimately also human infection (Ogada et al., 2012; Figure 3C) with health costs of up to US $1 billion annually in countries such as India (Markandya et al., 2008).

#### Soil resources and coastal erosion

Wetland vegetation provides critical ecosystem services, including erosion and flood control globally valued at US $14 trillion annually (de Groot et al., 2006). Studies have shown that overfishing in the Northeastern USA has reduced the abundances of predators of herbivorous crabs (*Sesarma reticulatum*). As a result, the crabs increase in abundance and over-feed on coastal cordgrass (*Spartina alterniflora*). Reduced cordgrass abundance in turn results in coastal erosion and flooding (Silliman and Bertness, 2002; Coverdale et al., 2013; Figure 3D).

### Cultural

#### Foundation species and biodiversity

Corals are foundation species that sustain hyper diverse communities and deliver important recreational (e.g., tourism) services worth at least US $36 billion annually (Spalding et al., 2017). These ecosystems are under threat from stressors such as overfishing, warming, or invasive species (Hughes et al., 2017), many times acting indirectly on coral health via interaction networks. For example, algae-eating fish prevent excessive algal growth on corals, which would otherwise kill corals and lead to the collapse of coral-associated fish communities (Jones et al., 2004; Hughes et al., 2007). Such balance has been broken in the Caribbean Sea due to the introduction of the invasive lionfish (*Pterois volitans*). This fish is a voracious predator that reduces populations of algivorous fishes, resulting in overgrowth of algae, declines in coral abundance, and dramatic reductions in overall coral reef diversity (Lesser and Slattery, 2011; Figure 3E).

#### Charismatic species

Numerous species of cultural importance are becoming rare or locally extinct due to the disruption of interaction networks within which these species are embedded. A prime example is the extinct Steller’s sea cows (*Hydrodamalis gigas*), which fed on kelp and lived in kelp forests in the Aleutian archipelago. While hunting was originally pointed as the main cause of its extinction, there is also evidence linking its decline to human impacts indirectly via effects on a third species. Surprisingly, overhunting of sea otters (*Enhydra lutris*) for the fur trade (Estes et al., 2016) increased their kelp-eating prey (e.g., sea urchins), decimating the kelp forests upon which sea cows depended for their survival (Figure 3F).

### Network management in action

**Figure 4.**
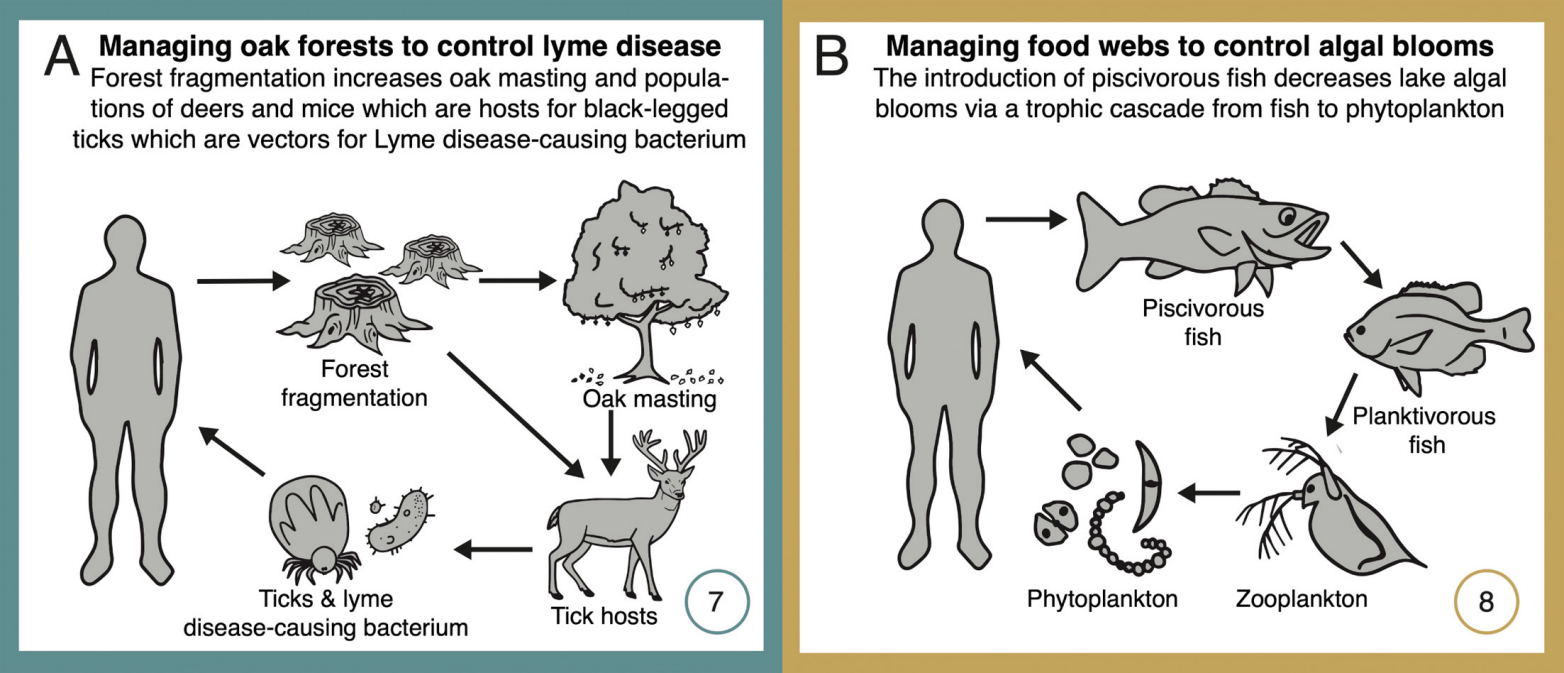
Network diagrams of systems using network management for disease prevention or forecasting (Lyme disease, **A**), and for trophic regulation of ecosystem productivity for fisheries and recreation (temperate lakes, **B**). See text for detailed explanations of each case study. Numbers (bottom right-hand corners) reference the depiction of these case studies in Figure 1. Some case studies address multiple classes of ecosystem service (as shown in Figure 1), but individual classes are highlighted here and in Figure 3 and in Appendix 1.

There are several case studies evidencing how an understanding of interaction networks can be effectively used to guide management and raise public awareness. In particular, here we draw from research and management in two study systems which serve as good examples in this regard (Figure 4).

#### Managing oak forest networks to control Lyme disease

Lyme disease is the costliest vector-borne disease in North America. In the United States alone, Lyme disease annually affects more than a half-million people (Adrion et al., 2015; Schwartz et al., 2017), with costs for healthcare exceeding US $1 billion (Adrion et al., 2015), and indirect costs from reduced recreation, tourism, and real estate values reaching $5 billion (Berry et al., 2018). More than two decades of research has revealed that the indirect effects of periodic ‘masting’ events – years of high acorn production – are key to understanding and managing Lyme disease risk. High acorn availability triggers increases in populations of white-footed mice (*Peromyscus leucopus*) and deer (*Odocoileus virginianus*), both critical hosts for black-legged ticks (*Ixodes scapularis*), which are vectors for the Lyme disease-causing bacterium (*Borrelia burgdorferi*) (Jones et al., 1998; Ostfeld et al., 2001). In contrast, increased diversity in non-competent hosts (thereby ‘diluting’ the availability of suitable hosts, particularly mice) reduces Lyme prevalence and thus risk to humans (Ostfeld and Keesing, 2000).

Both forest fragmentation and the loss of key predators, such as foxes and certain bird species, have disrupted the regulating services provided by this network, exacerbating disease risk (Allan et al., 2003; Levi et al., 2012). For instance, fragmented forests harbor larger populations of mice and deer, while simultaneously having lower predator densities, thus amplifying tick populations and the risk of disease transmission. Further, forest fragmentation also leads to increased acorn production (Morán-López et al., 2016), an additional pathway boosting mouse populations and disease risk. These insights have led to several innovative management strategies aimed at restoring balance in these networks. For instance, forest managers have implemented measures to increase habitat connectivity with corridors, reducing fragment edge and area effects, and have also reintroduced predators, practices that have jointly contributed to the recovery of predator populations and reduced mouse abundances (Levi et al., 2012; Ostfeld and Keesing, 2012). In addition, understanding the network dynamics between oak masting and disease risk has made it possible for predictive models to forecast Lyme disease risk 2 years in advance (Ostfeld et al., 2006), allowing health authorities and land managers to implement proactive interventions, including public awareness campaigns and targeted host population controls and application of insecticides in high-risk zones (Ostfeld and Keesing, 2000). The understanding gained through these long-term studies underscores both the complexity and value of managing interaction networks to sustain ecosystem services.

#### Managing aquatic food webs to control algal blooms

Harmful algal blooms in lakes cause massive economic losses in temperate regions (Carpenter et al., 2010; Cooke et al., 2016). Studies suggest that the economic costs in the United States alone could exceed $4 billion annually due to losses in fisheries, tourism, and public health impacts (Anderson et al., 2000). In these lakes, large piscivorous fish play a crucial role in regulating a well described four-level food chain including smaller fish that feed on grazing zooplankton (e.g., large species of water fleas, *Daphnia*) which in turn feed on algae (Carpenter et al., 2001). When populations of piscivorous fish decline, mainly due to overfishing, smaller zooplanktivorous fish proliferate unchecked, resulting in overconsumption of algae-consuming zooplankton (Lathrop et al., 2002). Due to weaker trophic control by zooplankton, algal populations proliferate, leading to algal blooms that can severely impact water quality by depleting oxygen levels, creating dead zones, and sometimes promoting harmful algal species that produce toxins detrimental to marine life and human health (Carpenter et al., 2010). Here, management actions have involved the reintroduction of top predators to reverse this food web imbalance. Piscivore reintroduction efforts have been successful in a number of regions and using different predatory fish (e.g., walleye, *Stizostedion vitreum*: Lathrop et al., 2002; largemouth bass, *Micropterus salmoides*: Mittelbach et al., 2006), leading to significant reductions in the populations of small predators and allowing algae-grazing organisms to flourish. This has ultimately led to improved water quality and a significant decrease in the occurrence of harmful algal blooms, effectively restoring lake food webs and associated ecosystem services across a broad range of biotic and abiotic conditions (Carpenter et al., 2010). Further, research has yielded a mechanistic understanding of the conditions under which these trophic cascades are strongest and manipulations are more likely to succeed (Hansson et al., 1998; Cooke et al., 2016), including the influence of additional stressors such as invasive species introductions (Walsh et al., 2016) or variability in nutrient inputs due to runoff (Carpenter et al., 2010), thereby guiding adaptive management.

### Technologies to accelerate network management

Technological advances are expanding our capacity to gather data on species interactions, elucidate networks, and unravel direct and indirect effects underlying ecosystem service changes (Figure 2). Here, we emphasize the contributions from imagery, telemetry and tracking, analysis of DNA, online environmental datasets, and computational techniques for data processing and analysis. All these tools provide novel data streams on species interactions that can be used by local managers to identify and monitor network changes affecting ecosystem services (Gray et al., 2014). This section is by no means exhaustive, but rather aimed to deliver a general view of how technological advances can propel network management drawing from a subset of useful examples.

DNA analyses of environment samples can be used to identify species based upon rapidly growing and publicly available databases on species’ DNA sequences such as the Barcode of Life Data System (BOLD), which now contains sequences from more than half a million species. For example, samples from animal guts or feces can reveal the diets of herbivores and predators (Kartzinel et al., 2015), samples of blood within blood-sucking parasites can reveal the identity of host species (Gogarten et al., 2020), while parasite samples can be used to identify the pathogens they harbor (e.g., bacteria, virus), revealing their role as disease vectors (Gangoso et al., 2019). Stable isotope analysis of animal and plant tissues is also used to characterize the diets of herbivores, seed dispersers, pollinators, and predators, and the sources of water and nutrients in plants (Boecklen et al., 2011). In addition, relatively simple and cheap approaches can be used to identify interactions as, for example, where the bite markings on clay caterpillars can assess the abundance of caterpillar predators (Roslin et al., 2017) or animal-marking dyes to reveal the species identities. Some of these methods have been coupled with molecular analyses as in the case of DNA analyses of saliva on clay caterpillars to identify attacking predators (Röbler et al., 2020).

Technologies for tracking movement are also being used to reveal species interactions. For example, tagged seeds can be located to understand the impacts of seed-dispersing animals (Jansen et al., 2012). Light-weight active devices also make it possible to track individual animals, including insects (Batsleer et al., 2020), while passive tags (or molecules) can be affixed to or inserted within animals to detect their presence at fixed detector locations (Stanley et al., 2021). Surveys of species presence and spatial distributions can also be used to infer interactions and are affordable with remotely operated fixed cameras (O’Connell et al., 2011), drones (Saunders et al., 2022), and even audio recordings (Wijers et al., 2021). For example, data on abundance and movement of animals that are pathogen reservoirs can be used to model rates of disease and parasite transmission to predict disease dynamics (Sah et al., 2017). Relatedly, statistical modeling of patterns of co-occurrences among multiple species can infer the interactions among those species (Zhu et al., 2019), and when such data are collected over time, fluctuations in the abundance of interacting species can be modeled to quantify interaction strength (Ye et al., 2015).

Citizen science efforts can also benefit from the use of technological tools, including immense quantities of imagery data that are now available from increasingly affordable remote autonomous cameras, drones, and smartphones, and satellites (Tibbetts, 2017; Verfuss et al., 2019). For example, the iNaturalist on-line app collects and identifies public smart phone images and currently includes 88 million geo-referenced observations of 344,000 species collected by nearly 2 million contributors from around the world (see 98). Similarly, the iDigBio Portal aggregates the digitized records from museum natural history collections and currently includes 130 million geo-referenced records. These geo-referenced biological datasets can in turn be combined with accessible, fine-scale environmental datasets on hydrology, geology, soils, and long-term daily climate records to accurately describe the physical setting in which species and species interactions are embedded.

Several areas of computer science are also potentiating our understanding of interaction networks and their responses to stressors, impacting the use and development of other technologies (as the above). Mathematical modeling can offer insights into the intricate relationships and interactions that shape ecological communities. For example, community dynamic models based on differential equations account for the interactions and interdependencies between multiple species and are used to understand and predict ecological networks (Thébault and Fontaine, 2010; Hu et al., 2022). These models enable a deeper understanding of how changes in one species can ripple through the network, affecting the entire ecosystem (Sentis et al., 2021; Arnoldi et al., 2022). Although they may oversimplify the complexities of real-world ecosystems, some of these limitations can be addressed using agent-based models that can serve as powerful tools for ecological network management with stakeholders by simulating complex interactions within ecosystems (McLane et al., 2011). Importantly, machine learning and other artificial intelligence tools will likely also significantly increase the speed and reliability of network analyses, both descriptive and predictive (Lucas, 2020). Published network datasets are ever increasing and offer abundant material for training machines and iterative processes of prediction testing, refinement, and further testing, and results can provide powerful insight into species interaction networks (reviewed by Pichler et al., 2020). For example, machine learning methods can make highly accurate predictions of species interactions and network structure (Pichler et al., 2020; Adhurya and Park, 2024) and can be used to predict effects of management (e.g., in agroecosystems; Ma et al., 2019) and anthropogenic stressors (Fricke et al., 2022) on networks.

In summary, the use of these emerging and complementary technologies will increase the robustness and availability of network-based information, which can then be used to increase the precision and effectiveness of interventions. Ultimately, this can lead to a more widespread adoption of network management.

### The process, challenge, and promise of implementing network management

**Figure 5.**
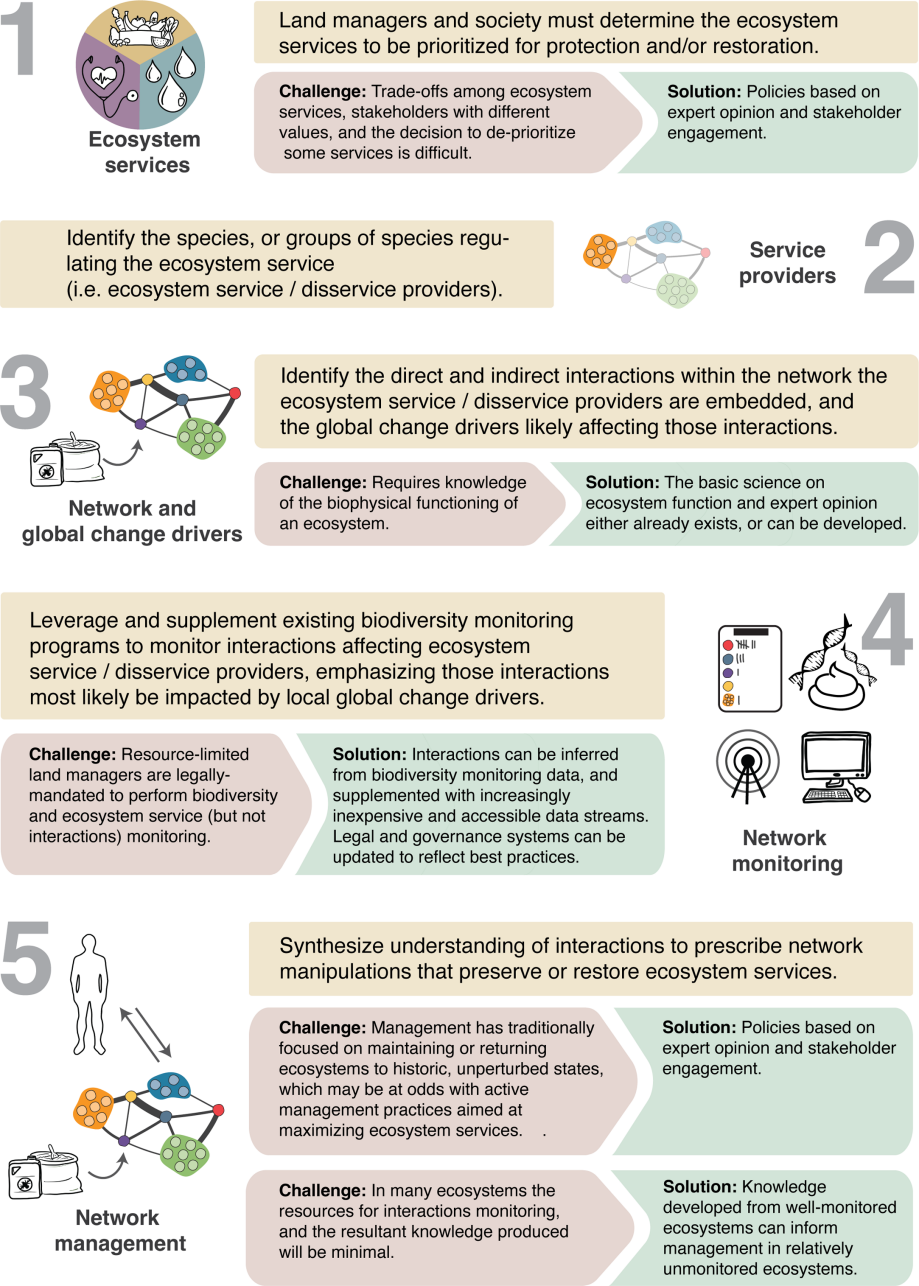
A general framework for developing network management. We outline the challenges to each of the five fundamental steps for transitioning to network management and suggest solutions in each case.

Harnessing novel streams of data on species interactions for network management will in turn require five steps, each of which is associated with its own set of unique challenges (Figure 5).

First is the need to identify the ecosystem services to be maximized or protected (Figure 5, step 1). One social challenge is that the perceived value of ecosystem services or disservices can vary among societal stakeholder groups (Gray et al., 2014), and trade-offs will have to be negotiated.

The next steps are to identify the species or guilds that provide or disrupt those identified ecosystem services (Timberlake et al., 2024), as well as networks of interactions that directly and indirectly affect these species’ or guild’s performance (Figure 5, steps 2 and 3). This requires knowledge on the basic science of ecosystem function, which exists in many cases or will need to be developed.

Monitoring programs can then be developed around focal interactions, focusing especially on those most likely to be disrupted by global change (step 4). Given manager resource limitations, such efforts will need to leverage the data produced from existing species monitoring programs, as well as citizen science data and other novel data streams (see technologies section). One added challenge is that current conservation laws and regulations typically require monitoring species, not interactions, and this will require the revisions to conservation law (Gray et al., 2014), including the establishment of common standards and currencies for interaction monitoring.

The final step in this process is to leverage the insights from network monitoring to prescribe active management that restores key interactions and networks that support targeted ecosystem services, including exporting knowledge gained from well-studied ecosystems to guide management of those for which less is known. Historic management practices have often taken the phenomenological approach of restoring ecosystems to historic biophysical conditions. In contrast, network management, with its focus on defined ecosystem services (step 1), will have to accept that such natural conditions are often unattainable, and that active manipulation (e.g., species introductions or removals) will thus be required to restore ecosystem services. This fact underscores the importance of clearly stated and accepted goals for the service to be maintained so that the benefits of such manipulations are understood (step 1). Such interventions will not only require knowledge of ecosystem function but will also require placing network management within an interdisciplinary framework (Bodin and Tengö, 2012; Felipe-Lucia et al., 2022), including the use of socio-ecological networks which analyze global change drivers from the perspective of human behaviors and social interaction networks influencing ecosystem management (Bodin et al., 2019; Yletyinen et al., 2021). The inclusion of indigenous knowledge within this context is also key (Ban et al., 2018).

Pursuing network management in turn offers three principal benefits. First, changes in interactions can provide early indications of ecosystem changes. Ecological surprises arise from lack of knowledge on indirect interactions within networks such that monitoring those interactions can provide information to forecast and better mitigate impacts. Second, each community has unique attributes and dynamics, so that monitoring interactions within each system provides knowledge on local responses to develop more effective system-specific interventions. Third, monitoring interactions can also yield generalities and promote synthesis. Institutionalizing network monitoring on a broad scale will fill key knowledge gaps, allowing managers and scientists to ask key questions such as: What types of interactions are most susceptible to change? What trophic levels are directly impacted by different agents of global change, and to which trophic levels do such effects propagate (i.e., indirect effects)? And finally, how do these dynamics vary among ecosystems and with respect to different agents of global change? Research shows common underlying network structures and subsets of ecosystem service providers with similar effects across ecosystems (Bramon Mora et al., 2018), and knowledge gained from well-studied systems (e.g., top-predator reintroductions, restoring foundation species) can be used in others that are less-studied. These cross-system commonalities and inferences can provide key knowledge for land managers and policy makers.

### Learning from agriculture

Although implementing network management will be challenging (Figure 5), its fundamental features can be observed in agricultural practices. Here, a single ecosystem service – crop yield – is often intentionally emphasized to guide management and monitoring activities (to the detriment of other services provided by the landscape), but societal priorities often also require a consideration of other services such as soil health, water quality, and biodiversity conservation (Tscharntke et al., 2005; Isbell et al., 2017; Figure 5, step 1). In addition, it is recognized that yield is affected by the negative direct effects of competing weeds and pest damage, direct and indirect costs of pesticide and herbicide application (Frank, 2024), and the indirect, beneficial effects of pollinators, detritivores, and predators that feed upon herbivorous insects (Figure 5, steps 2 and 3). An understanding of this network in turn informs interaction monitoring, including densities of competitors, herbivores, predators, and pollinators (Figure 5, step 4) (Eubanks and Finke, 2014; Vandermeer et al., 2019). Such monitoring is then leveraged to guide the manipulation of network structure to maximize desired ecosystem services (Figure 5, step 5). In addition, network management can occur at short timescales. For example, densities of herbivorous insects or weeds are monitored, and when they cross established economic thresholds – that is, the densities where the economic loss warrants intervention – pest control practices are deployed including the application of herbicides or insecticides or the use of beneficial microbes. Longer-term manipulations include the introduction of novel native predators and the breeding of plant resistance. Interestingly, some of these practices, for example, predator introductions, can be short term if the trophic dynamics have already been worked out (e.g., Rosenthal et al., 2021). Longer-term manipulations also include cultural practices to promote predator abundance and diversity, which maximizes control of agricultural pests while also promoting biodiversity conservation goals (Gurr et al., 2017). Finally, strategies that are developed within well-studied systems lead to a holistic understanding of best practices to guide network management in other, less studied agricultural systems (Figure 5, step 5).

Agriculture also demonstrates the capacity of technological advances to accelerate network management. Agricultural pests can now be monitored through sampling of herbivore-induced plant odors (Arce et al., 2024), spectral imaging (Näsi et al., 2015; Horgan et al., 2020), as well as acoustic sensors and cameras both from ground- and drone-based sensors (Weiss et al., 2020; Preti et al., 2021). Analysis of both above- and below-ground environmental DNA is used to assess both plant pests as well as beneficial mutualists (Bender et al., 2016; Kestel et al., 2022). All of these data streams are in turn assessed using artificial intelligence (e.g., machine learning insect image-based identification; Blair et al., 2020) for pest detection (Bjerge et al., 2023) and simulate management effects on interaction networks (Ma et al., 2019). By bridging scientific research, technological innovation, and societal priorities, agriculture serves as a proving ground for the sustainable management of ecological networks, with insights that extend far beyond the boundaries of farmland.

### Conclusion

Ecological science provides robust evidence for how the impacts of global change on human wellbeing arise unexpectedly due to complex chains of indirect species interactions. The very first step to avoid future detrimental ecological surprises will be to increase monitoring of species interactions. This knowledge can then improve our ability to effectively manipulate networks to sustain the production of ecosystem goods and services. Although network management is more complex than past approaches, new technological advances provide an unprecedented opportunity to develop network management as a best practice for maintaining the integrity of the biological systems underlying human prosperity.

## Figures and Tables

**Appendix 1—figure 1.**
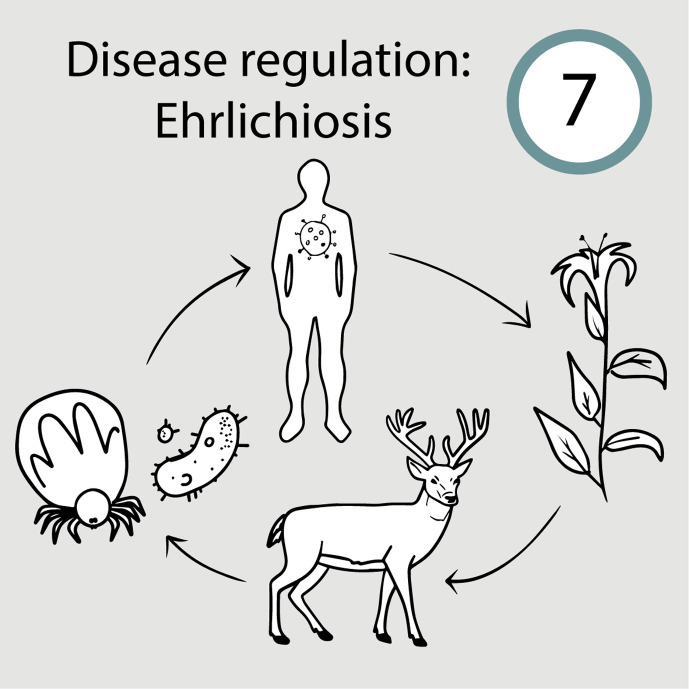
The risk of tick-borne diseases increases when deer congregate in patches of invasive honeysuckle (Case study 7 in manuscript Figure 1). Honeysuckle is an invasive woody shrub in the Northeastern United States that forms dense understory patches. Deer preferentially use these dense patches for increased shelter, protection from predators, and food (Allan et al., 2010). Deer are also the primary host for the lone star tick (*Amblyomma americanum*) and are a reservoir of bacterial pathogens. The high density of deer in honeysuckle patches results in a higher proportion of tick blood-meals from deer, and a greater prevalence of ticks infected with *Ehrlichia chaffeensis* (agents of human ehrlichiosis) (Allan et al., 2010). Therefore, the honeysuckle invasion ultimately increases the risk of human disease via altered interactions between plants, deer, ticks, and bacterial pathogens.

**Appendix 1—figure 2.**
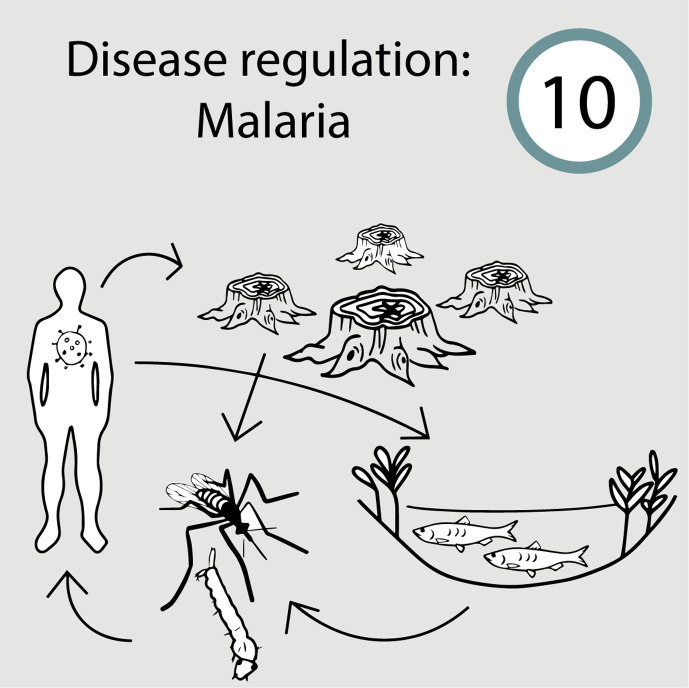
Malaria transmission rates increase as deforestation creates mosquito habitat (Case study 10 in manuscript Figure 1). Malaria infection has long been a critical health issue in Amazonian Brazil. There were 6 million cases annually in the 1940s, but control efforts were able to reduce cases to <50,000 by the early 1960s. Since then, there has been a resurgence of malaria largely driven by deforestation and land-use changes, altering mosquito–human interactions (MacDonald and Mordecai, 2019). Deforestation can increase infectious disease transmission by expanding the spatial overlap between people and wildlife disease reservoirs (Jones et al., 2008). In this case, not only are frontier settlements encroaching on mosquito habitat, but deforestation itself creates more mosquito breeding habitat near human settlements. The mosquitoes spreading malaria proliferate in larger ponds with more algae, leaf litter, and emergent grasses – conditions characteristic of fish farms created by settlers (Vittor et al., 2009). This case study exemplifies how environmental change affects human health, and how shifts in species interactions can mediate these effects.

**Appendix 1—figure 3.**
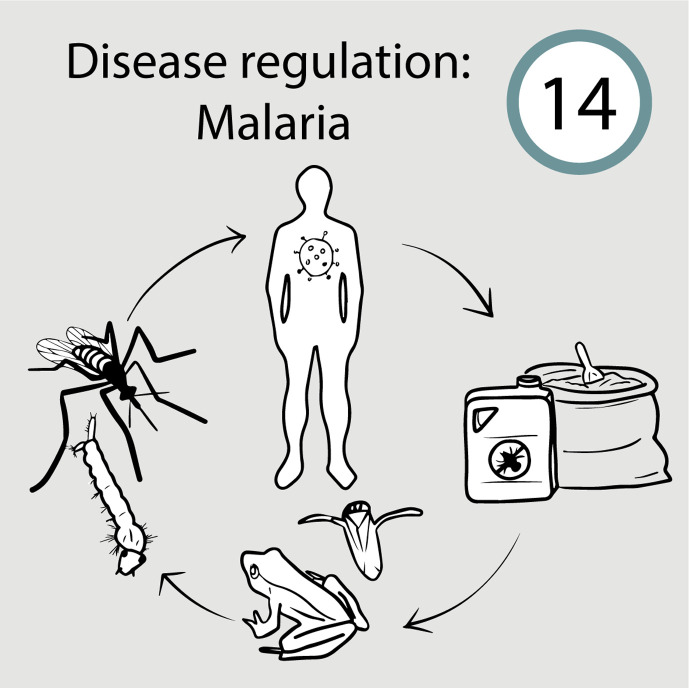
Insecticides can harm aquatic predators, indirectly increasing mosquito abundance and malaria transmission (Case study 14 in manuscript Figure 1). Efforts to control malaria, a threat to half of the world’s population (World Health Organization, 2020), can backfire when insecticides are used against malaria-transmitting mosquitoes. While these compounds may suppress mosquitoes directly, they also negatively affect fish and predatory insects that would otherwise control mosquito populations, paradoxically leading to mosquito and malaria outbreaks (Roux and Robert, 2019). This example demonstrates that disease regulation requires understanding how disease vectors are embedded in complex interaction networks that, if disrupted, can have large consequences for human health.

**Appendix 1—figure 4.**
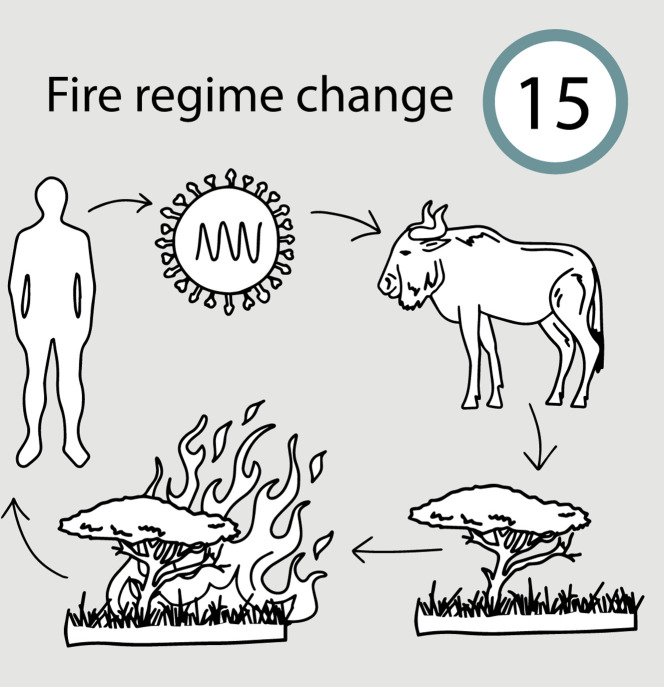
Eradication of rinderpest increased wildebeest abundance and grazing, reducing fire frequency, and increasing C storage (Case study 15 in manuscript Figure 1). Rinderpest is an infectious viral disease that infects ungulates. The virus arrived in sub-Saharan Africa in 1887 with the import of infected cattle. Following the introduction, rinderpest spread to domesticated and wild ungulates, causing widespread ungulate mortality and famine. In the 1960s, a successful vaccination program eradicated rinderpest. The abundance of many ungulates, particularly wildebeest, rapidly increased without this source of mortality. The increase in wildebeest grazing on the Serengeti reduced fuel loads for fires, decreased fire frequency, and increased tree density. This shift was so significant that the Serengeti switched from a carbon source to a carbon sink (Holdo et al., 2009).

**Appendix 1—figure 5.**
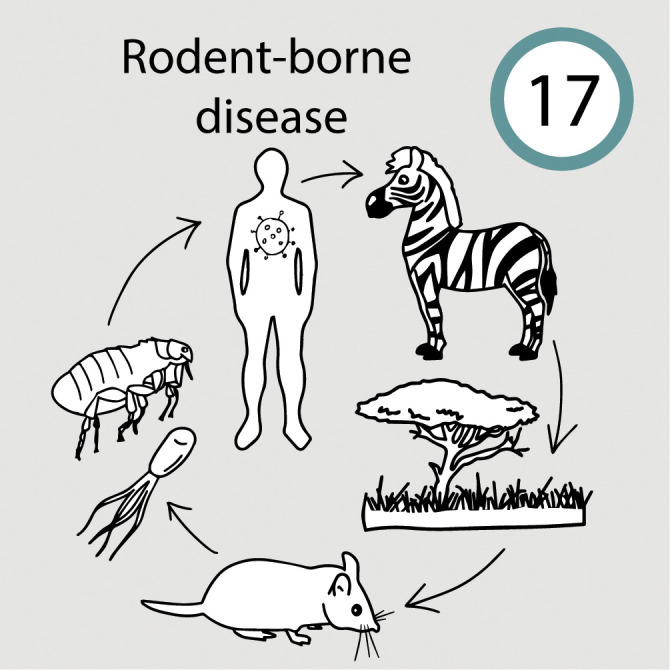
The extirpation of large wildlife alters vegetation, increasing the abundance of rodents and fleas and the risk of bartonellosis (Case study 17 in manuscript Figure 1). Large-bodied species are at a high risk of extinction, and their loss may have cascading impacts on other trophic levels (Ripple et al., 2017). Large herbivores are declining across African savannas due to land-use change and hunting. The loss of large herbivores has caused an increase in rodent populations through both direct and indirect pathways. Large herbivores directly compete with small mammals for food, so their extirpation reduces competition, allowing rodent populations to proliferate (Keesing, 2000). Simultaneously, their extirpation results in large vegetation structural changes that may also favor small mammals (Young et al., 2013). The increase in rodent abundance results in a higher abundance of their ectoparasites (e.g., fleas) and the pathogens they carry (e.g., Bartonella), ultimately increasing the risk of disease in humans (Young et al., 2014).

**Appendix 1—figure 6.**
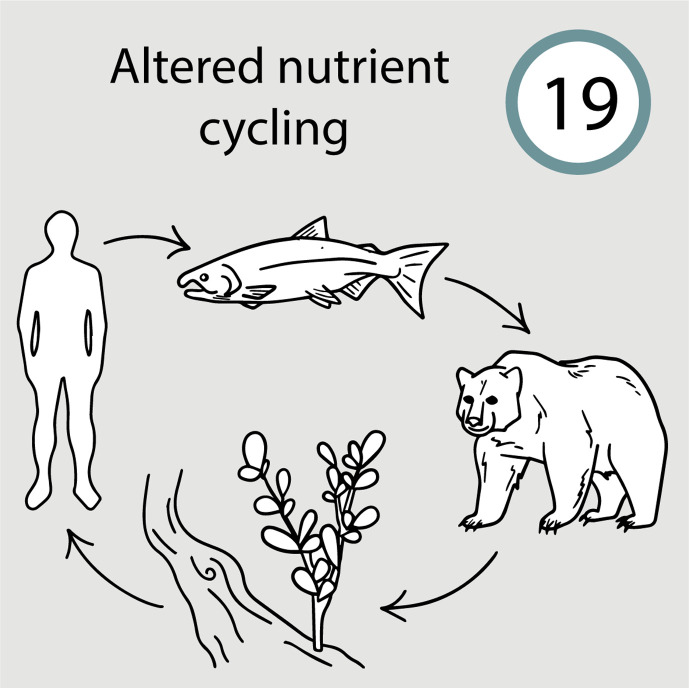
Overfishing salmon reduces soil nutrients due to the reduction of bear-mediated nutrient movement (Case study 19 in manuscript Figure 1). Climate change and overfishing reduce pacific salmon populations (*Oncorhynchus* spp.) with unexpected indirect effects on inland ecosystems. Migrating salmon spawn and die in inland rivers and streams, providing large nutrient inputs to adjacent forests from carcasses or feces from bears (*Ursus* spp.) and other salmon predators, such that declines in salmon populations are causing reductions in soil fertility and shifts in plant communities (Hocking and Reynolds, 2011). By recognizing these indirect effects, management practices are now starting to consider networks affecting ecosystem service or disservice providers that determine soil erosion, fertility, and other associated services.

**Appendix 1—figure 7.**
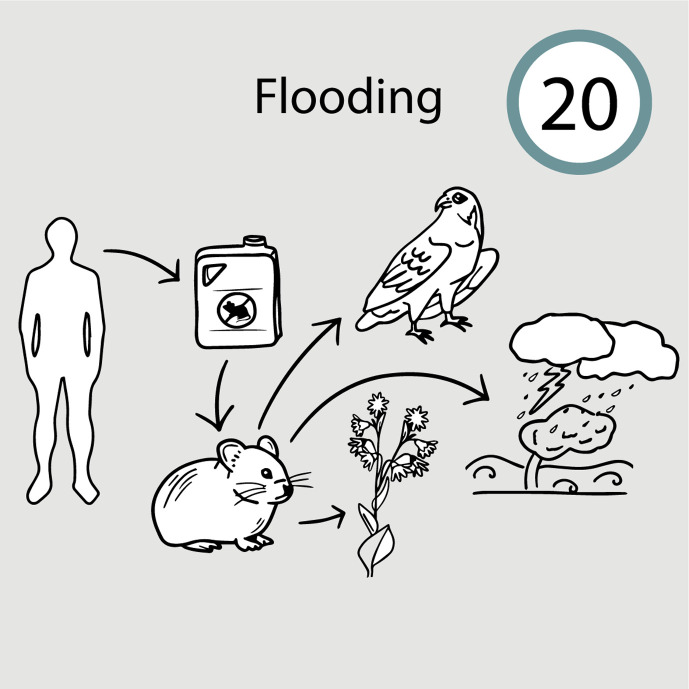
The poisoning of pikas leads to a loss of avian predators, changing soil water retention, and increasing flood severity (Case study 20 in manuscript Figure 1). The plateau pika (*Ochotona curzoniae*) is a keystone species and ecosystem engineer in the Qinghai–Tibetan Plateau region (Smith et al., 2019). The plateau pikas are important prey for avian and mammalian predators and dig burrows that nesting birds use. Burrowing also increases local plant diversity and soil water retention. Because pikas compete for food with local livestock, there have been on-going pika poisoning programs since the 1960s, poisoning pikas across hundreds of thousands of square kilometers at the cost of over $100 million USD. The loss of pika has had cascading impacts on other species and the ecosystem – their predators have been extirpated, plant diversity reduced, and soil water infiltration decreased. The eradication of the perceived pests has ultimately degraded cultural (biodiversity) and regulating (flood control) ecosystem services.

**Appendix 1—figure 8.**
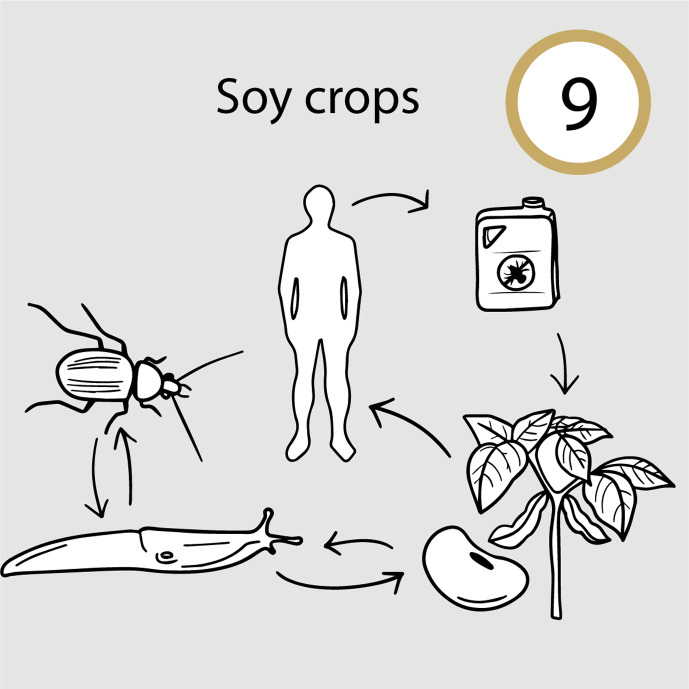
Insecticides applied to soybeans concentrate in slugs killing their predators ultimately increasing slug herbivory (Case study 9 in manuscript Figure 1). Agricultural practices, such as using insecticides, can alter species interactions affecting the services provided by agroecosystems. Neonicotinoids are the most common insecticide used across the globe to reduce pests in agricultural landscapes and increase crop yield. These insecticides are often coated onto seeds, protecting seeds and early stages of the plant as they are absorbed into tissues as the plant germinates and grows. Soybeans are one of the crops that routinely receive a neonicotinoid seed coating in North American agriculture. Although the goal of the insecticide application is to decrease herbivory, it has the opposite effect because of a series of species interactions. First, slugs eat neonicotinoids. They are not affected by the insecticide, and instead, the toxin is concentrated in their tissues. Next, slug predators are killed by the insecticide when they eat slugs, reducing the abundance of slug predators. The insecticides, therefore, increase herbivory by weakening pest biocontrol, ultimately reducing crop yields (Douglas et al., 2015).

**Appendix 1—figure 9.**
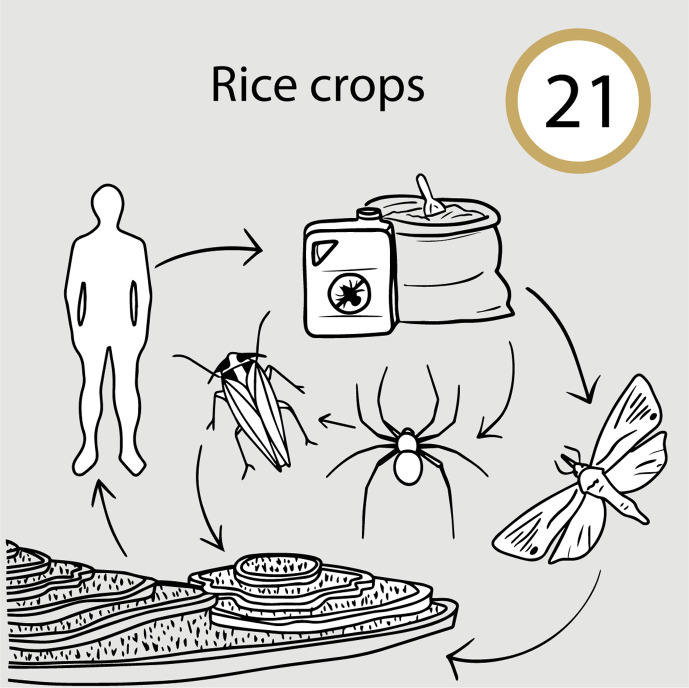
Pesticides applied to rice reduced competition and predation on a herbivore pest, increasing herbivore damage of rice (Case study 21 in manuscript Figure 1). In Indonesia, the use of pesticides against an economically important insect herbivore of rice, the yellow stem borer (*Scirpophaga incertulas*), unexpectedly created an even greater pest problem. Paradoxically, the suppression of the stem borer boosted populations of a secondary pest, the brown planthopper (*Nilaparvata lugens*), which is less susceptible to pesticides. Insecticides benefited the brown plant hopper by reducing competition with the stem borer, and by killing predatory arthropods that had previously held it in check (Heinrichs and Mochida, 1984; Settle et al., 1996). Such unintended impacts of insecticides are leading to agricultural practices that reduce pesticide use in favor of practices that seek to conserve networks of interactions that sustain these ecosystem service providers.

**Appendix 1—figure 10.**
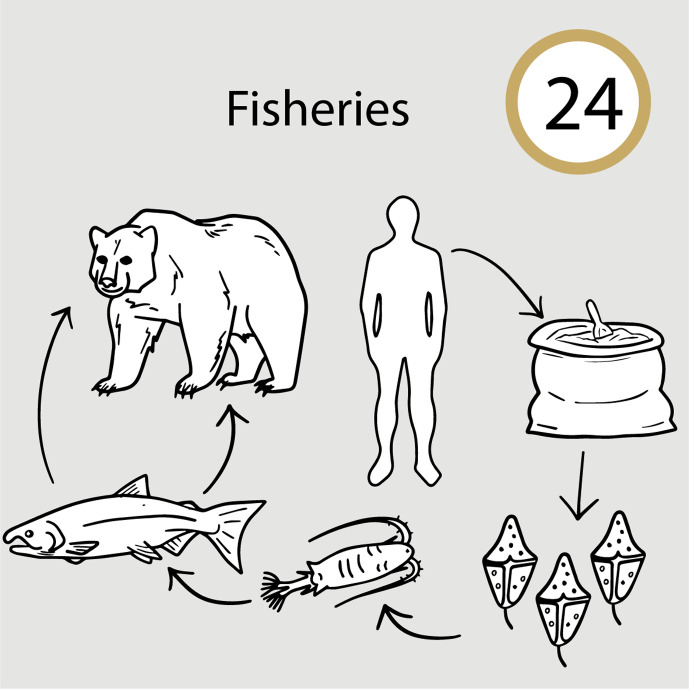
Terrestrial, marine mammals, and fish die when they consume prey containing toxins produced by fertilizer-fueled algal blooms (Case study 24 in manuscript Figure 1). Humans have dramatically accelerated nitrogen (Galloway et al., 2008) and phosphorus (Van Vuuren et al., 2010) cycles. Nutrients are transported by runoff water from terrestrial systems into lakes and rivers (Carpenter et al., 1998), eventually making their way to coastal ecosystems and oceans (Howarth et al., 1996). Upon reaching waterways, nutrients promote excessive plant growth and algal blooms (i.e., cultural eutrophication) which can impact aquatic ecosystems in various ways. Nutrient inputs increase the prevalence of harmful algal blooms that produce high levels of phycotoxins that affect organisms at higher trophic levels (Anderson et al., 2012). Toxic algae are consumed by zooplankton which are then fed upon by higher trophic level organisms such as fish. Fish may be directly killed by phycotoxins, or they may survive and in turn kill mammals and birds that prey on these fish (Landsberg, 2002). These findings teach us that to mitigate eutrophication effects on ecosystem function, we need to understand how its effects are transmitted through food webs via species interactions. Doing so will provide the scientific basis for protecting aquatic food webs, conserving and managing species of economic importance, and improving human wellbeing.

**Appendix 1—figure 11.**
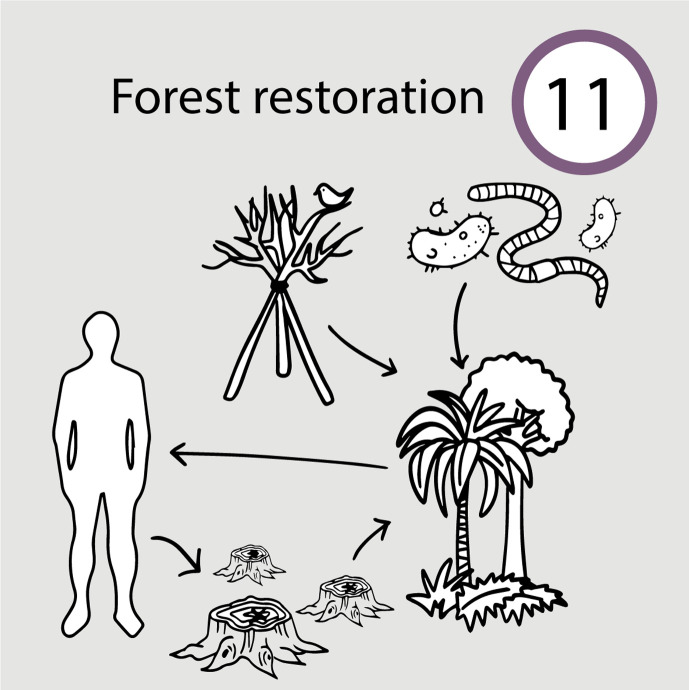
Rainforest restoration benefits from perches for birds and bats, shelters for reptiles and small mammals, and topsoil containing microbes, earthworms, and fungi (Case study 11 in manuscript Figure 1). Tropical rainforests have experienced deforestation for decades. Conservation efforts are increasingly focused on restoration efforts, turning deforested areas back into diverse and functioning ecosystems that provide many ecosystem services (e.g., biodiversity, species used in medicine). The most common restoration technique is creating tree plantations. Plantations vary in how successful they are at restoring fully functional ecosystems (Guariguata and Ostertag, 2001). Instead, techniques that consider species interactions and use a network approach have been more predictably successful (Bechara et al., 2016). One such approach is nucleation, where animals that directly and indirectly interact with plants are added in addition to planting trees. Successful additions include (1) Soil relocation from natural remnants to reestablish edaphic communities; (2) Seed banks from natural forest remnants; (3) Artificial perches for birds to use; (4) Artificial shelters for reptiles and small mammals. By nucleating the recovery of species that interact with rainforest plants, the ecosystem is able to restore ecosystem services more frequently and quickly than those that ignore species interactions (Bechara et al., 2016).

**Appendix 1—figure 12.**
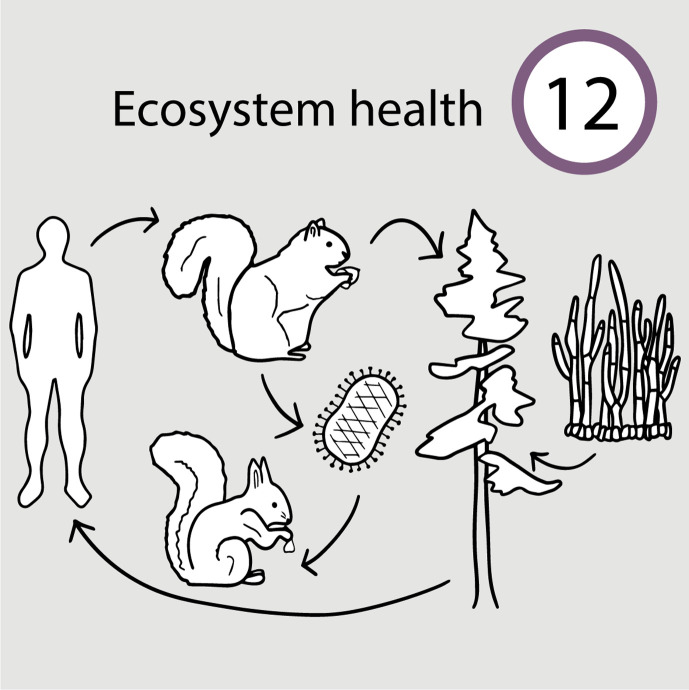
Invasive gray squirrels out-compete and infect native red squirrels and increase fungal infections on timber trees through bark peeling (Case study 12 in manuscript Figure 1). Eastern gray squirrels are invasive across Europe, having been introduced from the Americas. Eastern gray squirrels thrive in oak and beech forest and urban environments and have been outcompeting the native red squirrel for habitat and food. Eastern gray squirrels may also be the source of a disease caused by parapoxvirus that kills native squirrels. The introduction of gray squirrels not only threatens the survival of native red squirrels but is a problem for commercial hardwood timber production (Bruemmer et al., 2000). Eastern gray squirrels pull the bark off trees resulting in wounds that severely decrease the value of the wood. The wound sites are also susceptible to fungal infections which can kill the trees (Bruemmer et al., 2000). Therefore, the invasive Eastern gray squirrel affects ecosystem services via altered species interactions, decreasing native biota (cultural ecosystem service), and negatively affecting the timber industry (provisioning service).

**Appendix 1—figure 13.**
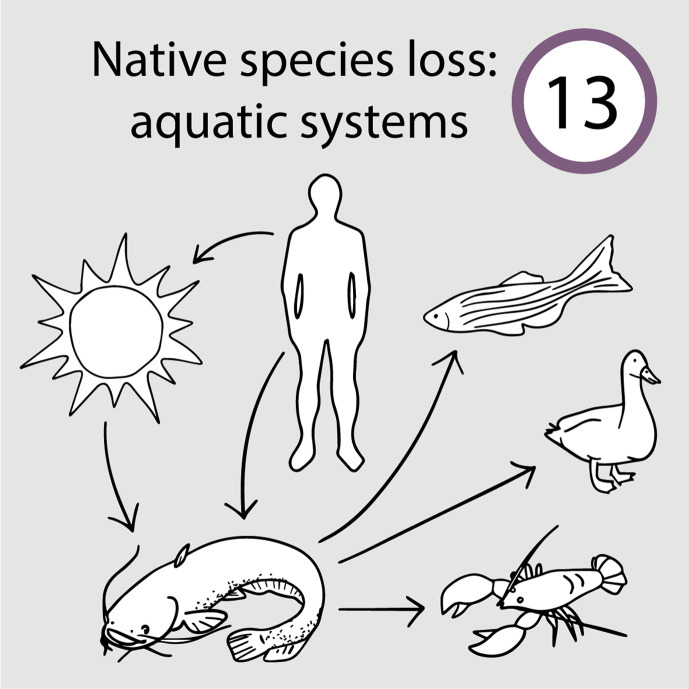
Alien fish species in Southern European rivers reduce native waterbirds, fish, and invertebrates, and are spreading as the climate warms (Case study 13 in manuscript Figure 1). The European catfish (*Silurus glanis*) is the largest-bodied freshwater fish of Europe and has a broad diet based mainly on other smaller fishes. It has been introduced to many areas in Europe outside its native range, mainly for recreational purposes (fishing) and is now posing a threat to local stream and river ecosystems across many countries and the services they provide (Copp et al., 2009). By feeding on native fishes, it drives changes in the structure of aquatic food webs, including changes in species composition and declines in abundance of both native vertebrate and invertebrate species (Carol et al., 2009). Impacts also include negative direct (and potentially also indirect) effects on waterbirds and other terrestrial species associated with riparian systems (Carol et al., 2009). Further, because this species is more prone to invade and successfully establish under warmer conditions, climate change-driven warming favors its invasion into southern regions such as the Mediterranean (Britton et al., 2010), amplifying the local strength and spatial extent of its ecological impacts.

**Appendix 1—figure 14.**
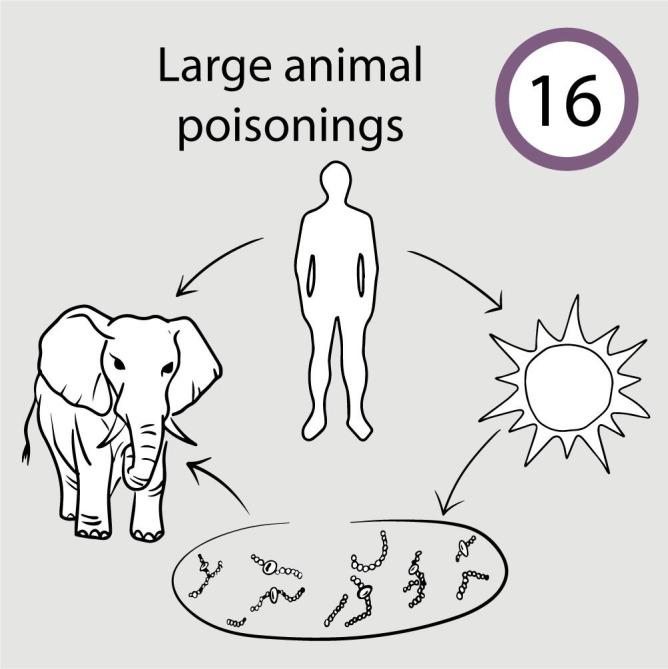
Poisonous microalgal blooms caused by warming can kill large animals at watering holes (Case study 16 in manuscript Figure 1). Botswana is home to one of the largest populations of elephants and is considered one of the last strongholds for this declining species. Elephant abundances have rapidly declined from an estimated 10 million in the 1930s to <500 thousand today due to poaching and land-use change. Close to 400 elephants were found dead between March and June 2020 near watering holes (Veerman et al., 2022). Toxins produced by cyanobacterial blooms were the culprit, with synchronous blooms across the Okavango Delta region in Botswana for many months beginning in 2019 (Veerman et al., 2022). Such blooms are promoted by low precipitation (longer water residence times), and high temperatures – both of which occurred in 2019/2020 in this region. This is not the first time cyanobacterial blooms have killed large charismatic and endangered species at watering holes (Oberholster et al., 2009), and it is unlikely to be the last, as climate change continues to alter precipitation and increase temperatures promoting cyanobacterial dominance in freshwaters worldwide (Paerl and Huisman, 2008).

**Appendix 1—figure 15.**
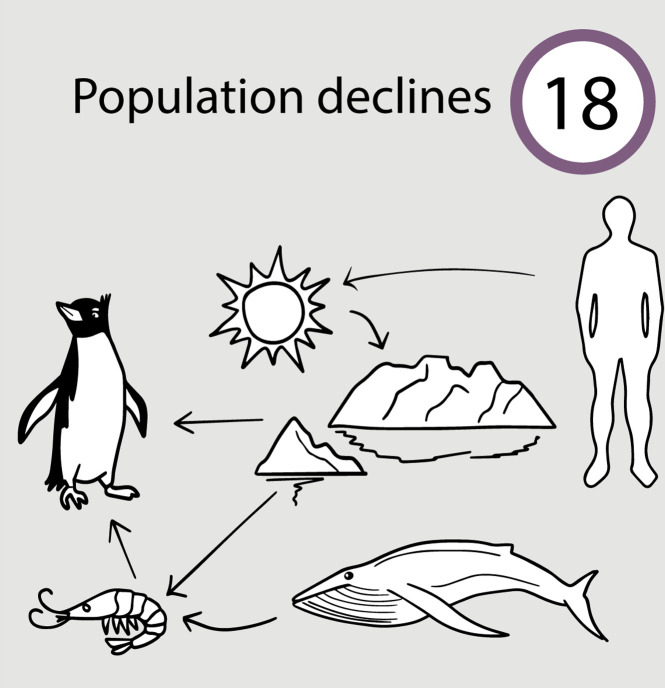
Climate warming has reduced penguin population abundance via shifts in prey biomass (Case study 18 in manuscript Figure 1). Penguin (*Pygoscelis adeliae*, *P. antarctica*) populations in Antarctica have suffered up to 50% reductions due to the indirect effects of climate warming. Krill (small crustaceans), the main food source for penguins (Trivelpiece et al., 2011), feed upon the algae that dwell under ice sheets, which also provides the krill physical shelter. Increasing ocean temperatures have reduced ice sheet coverage, reducing krill abundance and thus affecting penguins, as well as other species such as whales, seals, and other sea birds (Piñones and Fedorov, 2016; Trivelpiece et al., 2011).

**Appendix 1—figure 16.**
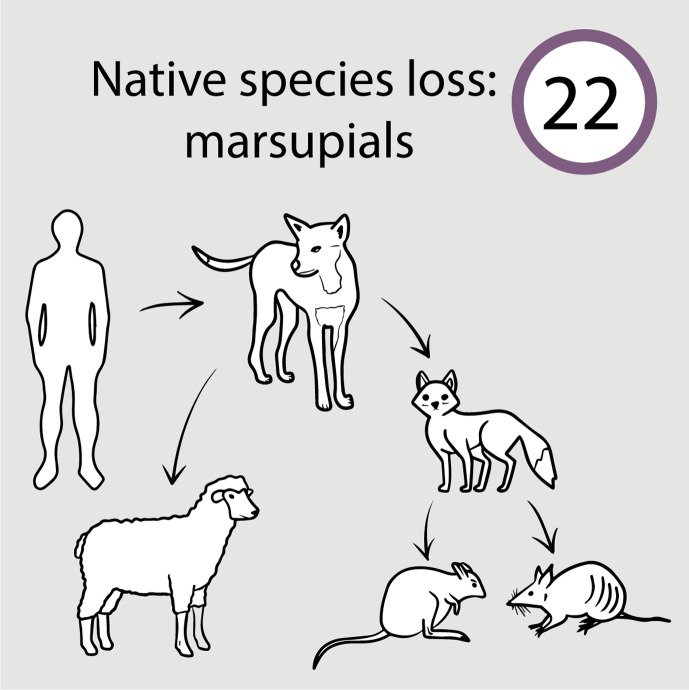
Australia’s marsupials were extirpated when dingo removal increased the abundance of invasive mesopredators (Case study 22 in manuscript Figure 1). Concomitant with the hominid invasion, Australia lost all of its native, large-bodied (>5 kg), terrestrial predators, resulting in one of the world’s most depleted predator guilds (Johnson et al., 2007), including its largest native predator, the dingo (*Canis lupus dingo*). Habitat loss and hunting as a preventive measure to protect sheep herds (without prior evidence that dingoes posed a significant risk to herds), drove the eventual elimination of the dingo and unexpected consequences due to altered species interactions. The loss of the dingo led to the rise of its former prey – non-native mid-sized predators such as the red fox (*Vulpes vulpes*) and feral cats (*Felis catus*). Mid-sized predators, left unchecked, unleashed drastic population declines and extinctions of their native marsupial prey throughout Australia. This wave of marsupial extinctions was symptomatic of underlying changes in species interactions due to top-predator extirpations (Letnic et al., 2012).

**Appendix 1—figure 17.**
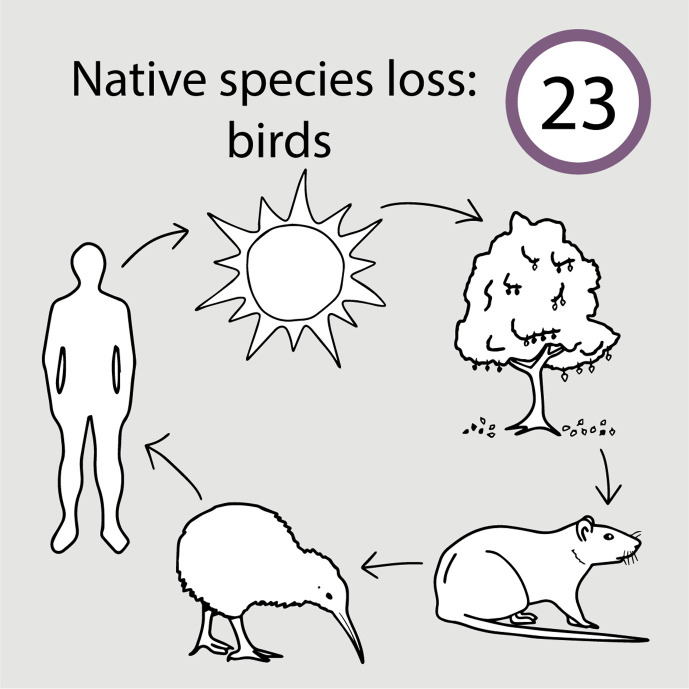
The proliferation of mice and rats following beech tree masting increases invasive stoat predation on native New Zealand birds (Case study 23 in manuscript Figure 1). Half of New Zealand’s endemic bird species have gone extinct since human colonization of the island, and many are currently threatened (Holdaway, 1999). The introduction of bird predators such as stoats has been a major driver of bird decline (Dilks et al., 2014). A cascade of species interactions magnifies the impact of invasive predators on native bird populations. First, beech trees reproduce through masting, simultaneously releasing flowers and seeds at irregular intervals (3–5 years, Schauber et al., 2002). Second, this large pulse of resources increases the population sizes of mice and rats and the population size of their predators – the invasive stoats (Dilks et al., 2014). The increased abundance of stoats is one of the most important factors driving forest birds' decline in New Zealand (Dilks et al., 2014; Wilson et al., 1998). As temperatures rise, seed production during masting is likely to increase (Schauber et al., 2002), with the greatest impact on predator abundance, not plant fitness (Bogdziewicz et al., 2020), exacerbating the reduction of native bird populations.

## Appendix 1

Additional examples of degraded ecosystem services driven by species interactions case studies depicted in Figure 1 that are not highlighted in the main text are described below with network diagrams. Numbers reference the depiction of these case studies in Figure 1. Some case studies address multiple classes of ecosystem service.

## References

[bib1] Adhurya S, Park YS (2024). A novel method for predicting ecological interactions with an unsupervised machine learning algorithm. Methods in Ecology and Evolution.

[bib2] Adrion ER, Aucott J, Lemke KW, Weiner JP (2015). Health care costs, utilization and patterns of care following Lyme disease. PLOS ONE.

[bib3] Allan BF, Keesing F, Ostfeld RS (2003). Effect of forest fragmentation on lyme disease risk. Conservation Biology.

[bib4] Allan BF, Dutra HP, Goessling LS, Barnett K, Chase JM, Marquis RJ, Pang G, Storch GA, Thach RE, Orrock JL (2010). Invasive honeysuckle eradication reduces tick-borne disease risk by altering host dynamics. PNAS.

[bib5] Anderson DM, Hoagland P, Kaoru Y, White AW, Anderson DM, Hoagland P, Kaoru Y, White AW (2000). Environmental and Resource Economics.

[bib6] Anderson DM, Cembella AD, Hallegraeff GM (2012). Progress in understanding harmful algal blooms: paradigm shifts and new technologies for research, monitoring, and management. Annual Review of Marine Science.

[bib7] Angelini C, Altieri AH, Silliman BR, Bertness MD (2011). Interactions among foundation species and their consequences for community organization, biodiversity, and conservation. BioScience.

[bib8] Arce CCM, Mamin M, Röder G, Kanagendran A, Degen T, Defossez E, Rasmann S, Akiyama T, Minami K, Yoshikawa G, Lopez-Hilfiker F, Cappellin L, Turlings TCJ (2024). Odor-based real-time detection and identification of pests and diseases attacking crop plants. bioRxiv.

[bib9] Arnoldi JF, Barbier M, Kelly R, Barabás G, Jackson AL (2022). Invasions of ecological communities: hints of impacts in the invader’s growth rate. Methods in Ecology and Evolution.

[bib10] Ban NC, Frid A, Reid M, Edgar B, Shaw D, Siwallace P (2018). Incorporate Indigenous perspectives for impactful research and effective management. Nature Ecology & Evolution.

[bib11] Barnes AD, Scherber C, Brose U, Borer ET, Ebeling A, Gauzens B, Giling DP, Hines J, Isbell F, Ristok C, Tilman D, Weisser WW, Eisenhauer N (2020). Biodiversity enhances the multitrophic control of arthropod herbivory. Science Advances.

[bib12] Bartomeus I, Saavedra S, Rohr RP, Godoy O (2021). Experimental evidence of the importance of multitrophic structure for species persistence. PNAS.

[bib13] Bascompte J, Stouffer DB (2009). The assembly and disassembly of ecological networks. Philosophical Transactions of the Royal Society of London. Series B, Biological Sciences.

[bib14] Batsleer F, Bonte D, Dekeukeleire D, Goossens S, Poelmans W, Van der Cruyssen E, Maes D, Vandegehuchte ML (2020). The neglected impact of tracking devices on terrestrial arthropods. Methods in Ecology and Evolution.

[bib15] Bechara FC, Dickens SJ, Farrer EC, Larios L, Spotswood EN, Mariotte P, Suding KN (2016). Neotropical rainforest restoration: comparing passive, plantation and nucleation approaches. Biodiversity and Conservation.

[bib16] Bender SF, Wagg C, van der Heijden MGA (2016). An underground revolution: biodiversity and soil ecological engineering for agricultural sustainability. Trends in Ecology & Evolution.

[bib17] Berry K, Bayham J, Meyer SR, Fenichel EP (2018). The allocation of time and risk of Lyme: A case of ecosystem service income and substitution effects. Environmental & Resource Economics.

[bib18] Bianco G, Manning P, Schleuning M (2024). A quantitative framework for identifying the role of individual species in Nature’s Contributions to People. Ecology Letters.

[bib19] Bjerge IK, Alison J, Dyrmann M, Frigaard CE, Mann HMR, Høye TT (2023). Accurate detection and identification of insects from camera trap images with deep learning. PLOS Sustainability and Transformation.

[bib20] Blair J, Weiser MD, Kaspari M, Miller M, Siler C, Marshall KE (2020). Robust and simplified machine learning identification of pitfall trap-collected ground beetles at the continental scale. Ecology and Evolution.

[bib21] Bodin Ö, Tengö M (2012). Disentangling intangible social–ecological systems. Global Environmental Change.

[bib22] Bodin Ö, Alexander SM, Baggio J, Barnes ML, Berardo R, Cumming GS, Dee L, Fischer AP, Fischer M, Mancilla-Garcia M, Guerrero A, Hileman J, Ingold K, Matous P, Morrison TH, Nohrstedt D, Pittman J, Robins G, Sayles J (2019). Improving network approaches to the study of complex social-ecological interdependencies. Nature Sustainability.

[bib23] Boecklen WJ, Yarnes CT, Cook BA, James AC (2011). On the use of stable isotopes in trophic ecology. Annual Review of Ecology, Evolution, and Systematics.

[bib24] Boerema A, Rebelo AJ, Bodi MB, Esler KJ, Meire P (2017). Are ecosystem services adequately quantified?. Journal of Applied Ecology.

[bib25] Bogdziewicz M, Kelly D, Thomas PA, Lageard JGA, Hacket-Pain A (2020). Climate warming disrupts mast seeding and its fitness benefits in European beech. Nature Plants.

[bib26] Bramon Mora B, Gravel D, Gilarranz LJ, Poisot T, Stouffer DB (2018). Identifying a common backbone of interactions underlying food webs from different ecosystems. Nature Communications.

[bib27] British Columbia Ministry of Forests, Lands and Natural Resource Operations (2012). British Columbia Ministry of Forests, Lands and Natural Resource Operations.

[bib28] Britton JR, Cucherousset J, Davies GD, Godard MJ, Copp GH (2010). Non‐native fishes and climate change: predicting species responses to warming temperatures in a temperate region. Freshwater Biology.

[bib29] Bruemmer C, Lurz P, Larsen K, Gurnell J (2000). Impacts and Management of the Alien Eastern Gray Squirrel in Great Britain and Italy.

[bib30] Buzhdygan OY, Meyer ST, Weisser WW, Eisenhauer N, Ebeling A, Borrett SR, Buchmann N, Cortois R, De Deyn GB, de Kroon H, Gleixner G, Hertzog LR, Hines J, Lange M, Mommer L, Ravenek J, Scherber C, Scherer-Lorenzen M, Scheu S, Schmid B, Steinauer K, Strecker T, Tietjen B, Vogel A, Weigelt A, Petermann JS (2020). Biodiversity increases multitrophic energy use efficiency, flow and storage in grasslands. Nature Ecology & Evolution.

[bib31] Carol J, Benejam LB, García-Berthou E (2009). Growth and diet of European catfish (*Silurus glanis*) in early and late invasion stages. Fundamental and Applied Limnology.

[bib32] Carpenter SR, Caraco NF, Correll DL, Howarth RW, Sharpley AN, Smith VH (1998). Nonpoint pollution of surface waters with phosphorus and nitrogen. Ecological Applications.

[bib33] Carpenter SR, Cole JJ, Hodgson JR, Kitchell JF, Pace ML, Bade D, Cottingham KL, Essington TE, Houser JN, Schindler DE (2001). Trophic cascades, nutrients, and lake productivity: whole-lake experiments. Ecological Monographs.

[bib34] Carpenter SR, Cole JJ, Kitchell JF, Pace ML, Terborgh J, Estes JR (2010). Trophic Cascades: Predators, Prey, and the Changing Dynamics of Nature.

[bib35] Cooke GD, Welch EB, Peterson S, Nichols SA (2016). Restoration and Management of Lakes and Reservoirs.

[bib36] Copp GH, Robert Britton J, Cucherousset J, García‐Berthou E, Kirk R, Peeler E, Stakėnas S (2009). Voracious invader or benign feline? A review of the environmental biology of European catfish Silurus glanis in its native and introduced ranges. Fish and Fisheries.

[bib37] Costanza R, d’Arge R, de Groot R, Farber S, Grasso M, Hannon B, Limburg K, Naeem S, O’Neill RV, Paruelo J, Raskin RG, Sutton P, van den Belt M (1997). The value of the world’s ecosystem services and natural capital. Nature.

[bib38] Costanza R, de Groot R, Sutton P, van der Ploeg S, Anderson SJ, Kubiszewski I, Farber S, Turner RK (2014). Changes in the global value of ecosystem services. Global Environmental Change.

[bib39] Coverdale TC, Herrmann NC, Altieri AH, Bertness MD (2013). Latent impacts: the role of historical human activity in coastal habitat loss. Frontiers in Ecology and the Environment.

[bib40] de Groot R, Stuip MAM, Finlayson CM, Davidson N (2006). Valuing wetlands: guidance for valuing the benefits derived from wetland ecosystem service Ramsar Technical Report no.3/CBD Technical Series no.27.

[bib41] Díaz S, Settele J, Brondízio ES, Ngo HT, Agard J, Arneth A, Balvanera P, Brauman KA, Butchart SHM, Chan KMA, Garibaldi LA, Ichii K, Liu J, Subramanian SM, Midgley GF, Miloslavich P, Molnár Z, Obura D, Pfaff A, Polasky S, Purvis A, Razzaque J, Reyers B, Chowdhury RR, Shin YJ, Visseren-Hamakers I, Willis KJ, Zayas CN (2019). Pervasive human-driven decline of life on earth points to the need for transformative change. Science.

[bib42] Dilks P, Willans M, Pryde M, Fraser I (2014). Large scale stoat control to protect mohua (*Mohoua ochrocephala*) and kaka (*Nestor meridionalis*) in the Eglinton Valley, Fiordland, New Zealand. New Zealand Journal of Ecology.

[bib43] Douglas MR, Rohr JR, Tooker JF (2015). Editor’s choice: Neonicotinoid insecticide travels through a soil food chain, disrupting biological control of non‐target pests and decreasing soya bean yield. Journal of Applied Ecology.

[bib44] Ellison AM (2019). Foundation species, non-trophic interactions, and the value of being common. iScience.

[bib45] Estes JA, Terborgh J, Brashares JS, Power ME, Berger J, Bond WJ, Carpenter SR, Essington TE, Holt RD, Jackson JBC, Marquis RJ, Oksanen L, Oksanen T, Paine RT, Pikitch EK, Ripple WJ, Sandin SA, Scheffer M, Schoener TW, Shurin JB, Sinclair ARE, Soulé ME, Virtanen R, Wardle DA (2011). Trophic downgrading of planet earth. Science.

[bib46] Estes JA, Brashares JS, Power ME (2013). Predicting and detecting reciprocity between indirect ecological interactions and evolution. The American Naturalist.

[bib47] Estes JA, Burdin A, Doak DF (2016). Sea otters, kelp forests, and the extinction of Steller’s sea cow. PNAS.

[bib48] Eubanks MD, Finke DL (2014). Interaction webs in agroecosystems: beyond who eats whom. Current Opinion in Insect Science.

[bib49] Felipe-Lucia MR, Guerrero AM, Alexander SM, Ashander J, Baggio JA, Barnes ML, Bodin Ö, Bonn A, Fortin M-J, Friedman RS, Gephart JA, Helmstedt KJ, Keyes AA, Kroetz K, Massol F, Pocock MJO, Sayles J, Thompson RM, Wood SA, Dee LE (2022). Conceptualizing ecosystem services using social–ecological networks. Trends in Ecology & Evolution.

[bib50] Filbee‐Dexter K, Pittman J, Haig HA, Alexander SM, Symons CC, Burke MJ (2017). Ecological surprise: concept, synthesis, and social dimensions. Ecosphere.

[bib51] Folke C, Polasky S, Rockström J, Galaz V, Westley F, Lamont M, Scheffer M, Österblom H, Carpenter SR, Chapin FS, Seto KC, Weber EU, Crona BI, Daily GC, Dasgupta P, Gaffney O, Gordon LJ, Hoff H, Levin SA, Lubchenco J, Steffen W, Walker BH (2021). Our future in the Anthropocene biosphere. Ambio.

[bib52] Frank KT, Petrie B, Choi JS, Leggett WC (2005). Trophic cascades in a formerly cod-dominated ecosystem. Science.

[bib53] Frank KT, Petrie B, Fisher JAD, Leggett WC (2011). Transient dynamics of an altered large marine ecosystem. Nature.

[bib54] Frank EG (2024). The economic impacts of ecosystem disruptions: costs from substituting biological pest control. Science.

[bib55] Fricke EC, Hsieh C, Middleton O, Gorczynski D, Cappello CD, Sanisidro O, Rowan J, Svenning J-C, Beaudrot L (2022). Collapse of terrestrial mammal food webs since the Late Pleistocene. Science.

[bib56] Galloway JN, Townsend AR, Erisman JW, Bekunda M, Cai Z, Freney JR, Martinelli LA, Seitzinger SP, Sutton MA (2008). Transformation of the nitrogen cycle: recent trends, questions, and potential solutions. Science.

[bib57] Gangoso L, Gutiérrez-López R, Martínez-de la Puente J, Figuerola J (2019). Louse flies of Eleonora’s falcons that also feed on their prey are evolutionary dead-end hosts for blood parasites. Molecular Ecology.

[bib58] Gogarten JF, Hoffmann C, Arandjelovic M, Sachse A, Merkel K, Dieguez P, Agbor A, Angedakin S, Brazzola G, Jones S, Langergraber KE, Lee K, Marrocoli S, Murai M, Sommer V, Kühl H, Leendertz FH, Calvignac‐Spencer S (2020). Fly‐derived DNA and camera traps are complementary tools for assessing mammalian biodiversity. Environmental DNA.

[bib59] Gray C, Baird DJ, Baumgartner S, Jacob U, Jenkins GB, O’Gorman EJ, Lu X, Ma A, Pocock MJO, Schuwirth N, Thompson M, Woodward G (2014). FORUM: Ecological networks: the missing links in biomonitoring science. The Journal of Applied Ecology.

[bib60] Guariguata MR, Ostertag R (2001). Neotropical secondary forest succession: changes in structural and functional characteristics. Forest Ecology and Management.

[bib61] Gurr GM, Wratten SD, Landis DA, You M (2017). Habitat management to suppress pest populations: progress and prospects. Annual Review of Entomology.

[bib62] Hairston NG, Smith FE, Slobodkin LB (1960). Community structure, population control, and competition. The American Naturalist.

[bib63] Hansson LA, Annadotter H, Bergman E, Hamrin SF, Jeppesen E, Kairesalo T, Luokkanen E, Nilsson PÅ, Søndergaard M, Strand J (1998). Minireview: biomanipulation as an application of food-chain theory: constraints, synthesis, and recommendations for temperate lakes. Ecosystems.

[bib64] Heinrichs EA, Mochida A (1984). From secondary to major pest status: the case of insecticide-induced rice brown planthopper, nilaparvata lugers, resurgence. Protection Ecology.

[bib65] Hekkala A-M, Kärvemo S, Versluijs M, Weslien J, Björkman C, Löfroth T, Hjältén J (2021). Ecological restoration for biodiversity conservation triggers response of bark beetle pests and their natural predators. Forestry.

[bib66] Hocking MD, Reynolds JD (2011). Impacts of salmon on riparian plant diversity. Science.

[bib67] Holdaway RN, MacPhee RDE (1999). Extinctions in Near Time: Causes, Contexts, and Consequences, Advances in Vertebrate Paleobiology.

[bib68] Holdo RM, Sinclair ARE, Dobson AP, Metzger KL, Bolker BM, Ritchie ME, Holt RD (2009). A disease-mediated trophic cascade in the serengeti and its implications for ecosystem C. PLOS Biology.

[bib69] Horgan FG, Jauregui A, Peñalver Cruz A, Crisol Martínez E, Bernal CC (2020). Changes in reflectance of rice seedlings during planthopper feeding as detected by digital camera: potential applications for high-throughput phenotyping. PLOS ONE.

[bib70] Howarth RW, Billen G, Swaney D, Townsend A, Jaworski N, Lajtha K, Downing JA, Elmgren R, Caraco N, Jordan T, Berendse F, Freney J, Kudeyarov V, Murdoch P, Zhao-Liang Z (1996). Regional nitrogen budgets and riverine N & P fluxes for the drainages to the North Atlantic Ocean: natural and human influences. Biogeochemistry.

[bib71] Hu J, Amor DR, Barbier M, Bunin G, Gore J (2022). Emergent phases of ecological diversity and dynamics mapped in microcosms. Science.

[bib72] Hughes TP, Rodrigues MJ, Bellwood DR, Ceccarelli D, Hoegh-Guldberg O, McCook L, Moltschaniwskyj N, Pratchett MS, Steneck RS, Willis B (2007). Phase shifts, herbivory, and the resilience of coral reefs to climate change. Current Biology.

[bib73] Hughes TP, Barnes ML, Bellwood DR, Cinner JE, Cumming GS, Jackson JBC, Kleypas J, van de Leemput IA, Lough JM, Morrison TH, Palumbi SR, van Nes EH, Scheffer M (2017). Coral reefs in the Anthropocene. Nature.

[bib74] Intergovernmental Science-Policy Platform on Biodiversity and Ecosystem Services (2024). Summary for Policymakers of the Assessment Report on the Interlinkages Among Biodiversity, Water, Food and Health (the Nexus Assessment).

[bib75] Isbell F, Adler PR, Eisenhauer N, Fornara D, Kimmel K, Kremen C, Letourneau DK, Liebman M, Polley HW, Quijas S, Scherer‐Lorenzen M (2017). Benefits of increasing plant diversity in sustainable agroecosystems. Journal of Ecology.

[bib76] Jansen PA, Hirsch BT, Emsens WJ, Zamora-Gutierrez V, Wikelski M, Kays R (2012). Thieving rodents as substitute dispersers of megafaunal seeds. PNAS.

[bib77] Johnson CN, Isaac JL, Fisher DO (2007). Rarity of a top predator triggers continent-wide collapse of mammal prey: dingoes and marsupials in Australia. Proceedings. Biological Sciences.

[bib78] Johnson CN, Balmford A, Brook BW, Buettel JC, Galetti M, Guangchun L, Wilmshurst JM (2017). Biodiversity losses and conservation responses in the anthropocene. Science.

[bib79] Jones CG, Lawton JH, Shachak M (1997). Positive and negative effects of organisms as physical ecosystem engineers. Ecology.

[bib80] Jones CG, Ostfeld RS, Richard MP, Schauber EM, Wolff JO (1998). Chain reactions linking acorns to gypsy moth outbreaks and Lyme disease risk. Science.

[bib81] Jones GP, McCormick MI, Srinivasan M, Eagle JV (2004). Coral decline threatens fish biodiversity in marine reserves. PNAS.

[bib82] Jones KE, Patel NG, Levy MA, Storeygard A, Balk D, Gittleman JL, Daszak P (2008). Global trends in emerging infectious diseases. Nature.

[bib83] Kartzinel TR, Chen PA, Coverdale TC, Erickson DL, Kress WJ, Kuzmina ML, Rubenstein DI, Wang W, Pringle RM (2015). DNA metabarcoding illuminates dietary niche partitioning by African large herbivores. PNAS.

[bib84] Keesing F (2000). Cryptic consumers and the ecology of an african savanna. BioScience.

[bib85] Kestel JH, Field DL, Bateman PW, White NE, Allentoft ME, Hopkins AJM, Gibberd M, Nevill P (2022). Applications of environmental DNA (eDNA) in agricultural systems: Current uses, limitations and future prospects. The Science of the Total Environment.

[bib86] Keyes AA, McLaughlin JP, Barner AK, Dee LE (2021). An ecological network approach to predict ecosystem service vulnerability to species losses. Nature Communications.

[bib87] Kremen C (2005). Managing ecosystem services: what do we need to know about their ecology?. Ecology Letters.

[bib88] Landsberg JH (2002). The effects of harmful algal blooms on aquatic organisms. Reviews in Fisheries Science.

[bib89] Lathrop RC, Johnson BM, Johnson TB, Vogelsang MT, Carpenter SR, Hrabik TR, Kitchell JF, Magnuson JJ, Rudstam LG, Stewart RS (2002). Stocking piscivores to improve fishing and water clarity: a synthesis of the Lake Mendota biomanipulation project. Freshwater Biology.

[bib90] Lawton JH, Gaston KJ, Lawton JH (2001). Encyclopedia of Biodiversity.

[bib91] Lesser MP, Slattery M (2011). Phase shift to algal dominated communities at mesophotic depths associated with lionfish (*Pterois volitans*) invasion on a bahamian coral reef. Biological Invasions.

[bib92] Letnic M, Fillios M, Crowther MS (2012). Could direct killing by larger dingoes have caused the extinction of the thylacine from mainland Australia?. PLOS ONE.

[bib93] Levi T, Kilpatrick AM, Mangel M, Wilmers CC (2012). Deer, predators, and the emergence of Lyme disease. PNAS.

[bib94] Lindenmayer DB, Likens GE (2010). The science and application of ecological monitoring. Biological Conservation.

[bib95] Lovett GM, Burns DA, Driscoll CT, Jenkins JC, Mitchell MJ, Rustad L, Shanley JB, Likens GE, Haeuber R (2007). Who needs environmental monitoring?. Frontiers in Ecology and the Environment.

[bib96] Lucas TCD (2020). A translucent box: interpretable machine learning in ecology. Ecological Monographs.

[bib97] Lynch HJ, Renkin RA, Crabtree RL, Moorcroft PR (2006). The influence of previous mountain pine beetle (dendroctonus ponderosae) activity on the 1988 yellowstone fires. Ecosystems.

[bib98] Ma A, Lu X, Gray C, Raybould A, Tamaddoni-Nezhad A, Woodward G, Bohan DA (2019). Ecological networks reveal resilience of agro-ecosystems to changes in farming management. Nature Ecology & Evolution.

[bib99] MacDonald AJ, Mordecai EA (2019). Amazon deforestation drives malaria transmission, and malaria burden reduces forest clearing. PNAS.

[bib100] Markandya A, Taylor T, Longo A, Murty MN, Murty S, Dhavala K (2008). Counting the cost of vulture decline—An appraisal of the human health and other benefits of vultures in India. Ecological Economics.

[bib101] McLane AJ, Semeniuk C, McDermid GJ, Marceau DJ (2011). The role of agent-based models in wildlife ecology and management. Ecological Modelling.

[bib102] Memmott J, Waser NM, Price MV (2004). Tolerance of pollination networks to species extinctions. Proceedings. Biological Sciences.

[bib103] Millennium Ecosystem Assessment (2005). Ecosystems and Human Well-Being.

[bib104] Mittelbach GG, Garcia EA, Taniguchi Y (2006). Fish reintroductions reveal smooth transitions between lake community states. Ecology.

[bib105] Morán-López T, Forner A, Flores-Rentería D, Díaz M, Valladares F (2016). Some positive effects of the fragmentation of holm oak forests: attenuation of water stress and enhancement of acorn production. Forest Ecology and Management.

[bib106] Myers RA, Hutchings JA, Barrowman NJ (1997). Why do fish stocks collapse? the example of cod in atlantic canada. Ecological Applications.

[bib107] Näsi R, Honkavaara E, Lyytikäinen-Saarenmaa P, Blomqvist M, Litkey P, Hakala T, Viljanen N, Kantola T, Tanhuanpää T, Holopainen M (2015). Using UAV-based photogrammetry and hyperspectral imaging for mapping bark beetle damage at tree-level. Remote Sensing.

[bib108] Nell CS, Abdala-Roberts L, Parra-Tabla V, Mooney KA (2018). Tropical tree diversity mediates foraging and predatory effects of insectivorous birds. Proceedings. Biological Sciences.

[bib109] Oberholster PJ, Myburgh JG, Govender D, Bengis R, Botha AM (2009). Identification of toxigenic microcystis strains after incidents of wild animal mortalities in the kruger national park, south africa. Ecotoxicology and Environmental Safety.

[bib110] O’Connell AF, Nichols JD, Karanth KU (2011). Camera Traps in Animal Ecology, Methods and Analyses.

[bib111] Ogada DL, Keesing F, Virani MZ (2012). Dropping dead: causes and consequences of vulture population declines worldwide. Annals of the New York Academy of Sciences.

[bib112] Ostfeld RS, Keesing F (2000). Biodiversity series: the function of biodiversity in the ecology of vector-borne zoonotic diseases. Canadian Journal of Zoology.

[bib113] Ostfeld RS, Jones CG, Wolff JO (2001). Of mice and mast: ecological connections in eastern deciduous forests. Bioscience.

[bib114] Ostfeld RS, Canham CD, Oggenfuss K, Winchcombe RJ, Keesing F (2006). Climate, deer, rodents, and acorns as determinants of variation in lyme-disease risk. PLOS Biology.

[bib115] Ostfeld RS, Keesing F (2012). Effects of host diversity on infectious disease. Annual Review of Ecology, Evolution, and Systematics.

[bib116] Paerl HW, Huisman J (2008). Blooms like it hot. Science.

[bib117] Paine RT (1969). The pisaster-tegula interaction: prey patches, predator food preference, and intertidal community structure. Ecology.

[bib118] Paine RT (1980). Food webs: linkage, interaction strength and community infrastructure. Journal of Animal Ecology.

[bib119] Paine RT, Tegner MJ, Johnson EA (1998). Compounded perturbations yield ecological surprises. Ecosystems.

[bib120] Pecl GT, Araújo MB, Bell JD, Blanchard J, Bonebrake TC, Chen IC, Clark TD, Colwell RK, Danielsen F, Evengård B, Falconi L, Ferrier S, Frusher S, Garcia RA, Griffis RB, Hobday AJ, Janion-Scheepers C, Jarzyna MA, Jennings S, Lenoir J, Linnetved HI, Martin VY, McCormack PC, McDonald J, Mitchell NJ, Mustonen T, Pandolfi JM, Pettorelli N, Popova E, Robinson SA, Scheffers BR, Shaw JD, Sorte CJB, Strugnell JM, Sunday JM, Tuanmu MN, Vergés A, Villanueva C, Wernberg T, Wapstra E, Williams SE (2017). Biodiversity redistribution under climate change: Impacts on ecosystems and human well-being. Science.

[bib121] Pejchar L, Mooney HA (2009). Invasive species, ecosystem services and human well-being. Trends in Ecology & Evolution.

[bib122] Pichler M, Boreux V, Klein MA, Schleuning M, Hartig F (2020). Machine learning algorithms to infer trait-matching and predict species interactions in ecological networks. Methods in Ecology and Evolution.

[bib123] Piñones A, Fedorov AV (2016). Projected changes of antarctic krill habitat by the end of the 21st century. Geophysical Research Letters.

[bib124] Pocock MJO, Evans DM, Memmott J (2012). The robustness and restoration of a network of ecological networks. Science.

[bib125] Preti M, Verheggen F, Angeli S (2021). Insect pest monitoring with camera-equipped traps: strengths and limitations. Journal of Pest Science.

[bib126] Ripple WJ, Wolf C, Newsome TM, Hoffmann M, Wirsing AJ, McCauley DJ (2017). Extinction risk is most acute for the world’s largest and smallest vertebrates. PNAS.

[bib127] Robbins ZJ, Xu C, Aukema BH, Buotte PC, Chitra-Tarak R, Fettig CJ, Goulden ML, Goodsman DW, Hall AD, Koven CD, Kueppers LM, Madakumbura GD, Mortenson LA, Powell JA, Scheller RM (2022). Warming increased bark beetle-induced tree mortality by 30% during an extreme drought in California. Global Change Biology.

[bib128] Röbler DC, Lötters S, Veith M, Fugmann M, Peters C, Künzel S, Krehenwinkel H (2020). An amplicon sequencing protocol for attacker identification from DNA traces left on artificial prey. Methods in Ecology and Evolution.

[bib129] Rosenthal SS, Campobasso G, Fornasari L, Sobhian R, Turner CE, James LF (2021). Noxious Range Weeds.

[bib130] Roslin T, Hardwick B, Novotny V, Petry WK, Andrew NR, Asmus A, Barrio IC, Basset Y, Boesing AL, Bonebrake TC, Cameron EK, Dáttilo W, Donoso DA, Drozd P, Gray CL, Hik DS, Hill SJ, Hopkins T, Huang S, Koane B, Laird-Hopkins B, Laukkanen L, Lewis OT, Milne S, Mwesige I, Nakamura A, Nell CS, Nichols E, Prokurat A, Sam K, Schmidt NM, Slade A, Slade V, Suchanková A, Teder T, van Nouhuys S, Vandvik V, Weissflog A, Zhukovich V, Slade EM (2017). Higher predation risk for insect prey at low latitudes and elevations. Science.

[bib131] Roux O, Robert V (2019). Larval predation in malaria vectors and its potential implication in malaria transmission: an overlooked ecosystem service?. Parasites & Vectors.

[bib132] Safranyik L, Carroll AL, Régnière J, Langor DW, Riel WG, Shore TL, Peter B, Cooke BJ, Nealis VG, Taylor SW (2010). Potential for range expansion of mountain pine beetle into the boreal forest of North America. The Canadian Entomologist.

[bib133] Sah P, Leu ST, Cross PC, Hudson PJ, Bansal S (2017). Unraveling the disease consequences and mechanisms of modular structure in animal social networks. PNAS.

[bib134] Saunders D, Nguyen H, Cowen S, Magrath M, Marsh K, Bell S, Bobruk J (2022). Radio-tracking wildlife with drones: a viewshed analysis quantifying survey coverage across diverse landscapes. Wildlife Research.

[bib135] Schauber EM, Kelly D, Turchin P, Simon C, Lee WG, Allen RB, Payton IJ, Wilson PR, Cowan PE, Brockie RE (2002). Masting by eighteen new zealand plant species: the role of temperature as a synchronizing cue. Ecology.

[bib136] Schmitz OJ, Weisser WW, Siemann E (2008). Insects and Ecosystem Function.

[bib137] Schwartz AM, Hinckley AF, Mead PS, Hook SA, Kugele KJ (2017). Surveillance for Lyme Disease — United States, 2008–2015.

[bib138] Sentis A, Montoya JM, Lurgi M (2021). Warming indirectly increases invasion success in food webs. Proceedings. Biological Sciences.

[bib139] Settle WH, Ariawan H, Astuti ET, Cahyana W, Hakim AL, Hindayana D, Lestari AS (1996). Managing tropical rice pests through conservation of generalist natural enemies and alternative prey. Ecology.

[bib140] Sguotti C, Otto SA, Frelat R, Langbehn TJ, Ryberg MP, Lindegren M, Durant JM, Chr Stenseth N, Möllmann C (2019). Catastrophic dynamics limit atlantic cod recovery. Proceedings. Biological Sciences.

[bib141] Silliman BR, Bertness MD (2002). A trophic cascade regulates salt marsh primary production. PNAS.

[bib142] Smith AT, Wilson MC, Hogan BW (2019). Functional-trait ecology of the plateau pika *Ochotona curzoniae* in the qinghai-tibetan plateau ecosystem. Integrative Zoology.

[bib143] Spalding M, Burke L, Wood SA, Ashpole J, Hutchison J, zu Ermgassen P (2017). Mapping the global value and distribution of coral reef tourism. Marine Policy.

[bib144] Stanley CQ, Dudash MR, Ryder TB, Shriver WG, Serno K, Adalsteinsson S, Marra PP (2021). Seasonal variation in habitat selection for a Neotropical migratory songbird using high‐resolution GPS tracking. Ecosphere.

[bib145] Thébault E, Fontaine C (2010). Stability of ecological communities and the architecture of mutualistic and trophic networks. Science.

[bib146] Tibbetts JH (2017). Remote sensors bring wildlife tracking to a new level: trove of data yields fresh insights – and challenges. Bioscience.

[bib147] Tilman D, Isbell F, Cowles JM (2014). Biodiversity and ecosystem functioning. Annual Review of Ecology, Evolution, and Systematics.

[bib148] Timberlake TP, Cirtwill AR, Sapkota S, Bhusal DR, Devkota K, Karki R, Joshi D, Saville NM, Kortsch S, Baral S, Roslin T, Memmott J (2024). Agricultural specialisation increases the vulnerability of pollination services for smallholder farmers. Journal of Applied Ecology.

[bib149] Timóteo S, Correia M, Rodríguez-Echeverría S, Freitas H, Heleno R (2018). Multilayer networks reveal the spatial structure of seed-dispersal interactions across the great rift landscapes. Nature Communications.

[bib150] Trivelpiece WZ, Hinke JT, Miller AK, Reiss CS, Trivelpiece SG, Watters GM (2011). Variability in krill biomass links harvesting and climate warming to penguin population changes in Antarctica. PNAS.

[bib151] Tscharntke T, Klein AM, Kruess A, Steffan‐Dewenter I, Thies C (2005). Landscape perspectives on agricultural intensification and biodiversity – ecosystem service management. Ecology Letters.

[bib152] Tylianakis JM, Didham RK, Bascompte J, Wardle DA (2008). Global change and species interactions in terrestrial ecosystems. Ecology Letters.

[bib153] United Nations Environment Programme (2022). Spreading like Wildfire – The Rising Threat of Extraordinary Landscape Fires.

[bib154] Vandermeer J, Armbrecht I, de la Mora A, Ennis KK, Fitch G, Gonthier DJ, Hajian-Forooshani Z, Hsieh HY, Iverson A, Jackson D, Jha S, Jiménez-Soto E, Lopez-Bautista G, Larsen A, Li K, Liere H, MacDonald A, Marin L, Mathis KA, Monagan I, Morris JR, Ong T, Pardee GL, Rivera-Salinas IS, Vaiyda C, Williams-Guillen K, Yitbarek S, Uno S, Zemenick A, Philpott SM, Perfecto I (2019). The community ecology of herbivore regulation in an agroecosystem: lessons from complex systems. BioScience.

[bib155] Van Vuuren DP, Bouwman AF, Beusen AHW (2010). Phosphorus demand for the 1970–2100 period: a scenario analysis of resource depletion. Global Environmental Change.

[bib156] Veerman J, Kumar A, Mishra DR (2022). Exceptional landscape-wide cyanobacteria bloom in okavango delta, botswana in 2020 coincided with a mass elephant die-off event. Harmful Algae.

[bib157] Verfuss UK, Aniceto AS, Harris DV, Gillespie D, Fielding S, Jiménez G, Johnston P, Sinclair RR, Sivertsen A, Solbø SA, Storvold R, Biuw M, Wyatt R (2019). A review of unmanned vehicles for the detection and monitoring of marine fauna. Marine Pollution Bulletin.

[bib158] Vittor AY, Pan W, Gilman RH, Tielsch J, Glass G, Shields T, Sánchez-Lozano W, Pinedo VV, Salas-Cobos E, Flores S, Patz JA (2009). Linking deforestation to malaria in the Amazon: characterization of the breeding habitat of the principal malaria vector, Anopheles darlingi. American Journal of Tropical Medicine and Hygiene.

[bib159] von Döhren P, Haase D (2015). Ecosystem disservices research: a review of the state of the art with a focus on cities. Ecological Indicators.

[bib160] Walsh JR, Carpenter SR, Vander Zanden MJ (2016). Invasive species triggers a massive loss of ecosystem services through a trophic cascade. PNAS.

[bib161] Waring RH, Pitman GB (1985). Modifying lodgepole pine stands to change susceptibility to mountain pine beetle attack. Ecology.

[bib162] Weiss M, Jacob F, Duveiller G (2020). Remote sensing for agricultural applications: a meta-review. Remote Sensing of Environment.

[bib163] Wijers M, Trethowan P, Du Preez B, Chamaillé-Jammes S, Loveridge AJ, Macdonald DW, Markham A (2021). Vocal discrimination of African lions and its potential for collar-free tracking. Bioacoustics.

[bib164] Wilson PR, Karl BJ, Toft RJ, Beggs JR, Taylor RH (1998). The role of introduced predators and competitors in the decline of Kaka (*Nestor meridionalis*) populations in New Zealand. Biological Conservation.

[bib165] Wootton JT, Emmerson M (2005). Measurement of interaction strength in nature. Annual Review of Ecology, Evolution, and Systematics.

[bib166] World Health Organization (2020). World Malaria Report. https://www.who.int/publications/i/item/9789240015791.

[bib167] Ye H, Deyle ER, Gilarranz LJ, Sugihara G (2015). Distinguishing time-delayed causal interactions using convergent cross mapping. Scientific Reports.

[bib168] Yletyinen J, Perry GLW, Stahlmann-Brown P, Pech R, Tylianakis JM (2021). Multiple social network influences can generate unexpected environmental outcomes. Scientific Reports.

[bib169] Young HS, McCauley DJ, Helgen KM, Goheen JR, Otárola-Castillo E, Palmer TM, Pringle RM, Young TP, Dirzo R (2013). Effects of mammalian herbivore declines on plant communities: observations and experiments in an African savanna. The Journal of Ecology.

[bib170] Young HS, Dirzo R, Helgen KM, McCauley DJ, Billeter SA, Kosoy MY, Osikowicz LM, Salkeld DJ, Young TP, Dittmar K (2014). Declines in large wildlife increase landscape-level prevalence of rodent-borne disease in Africa. PNAS.

[bib171] Zhu C, Gravel D, He F (2019). Seeing is believing? comparing plant-herbivore networks constructed by field co-occurrence and DNA barcoding methods for gaining insights into network structures. Ecology and Evolution.

